# New insights into the electrochemical behavior of acid orange 7: Convergent paired electrochemical synthesis of new aminonaphthol derivatives

**DOI:** 10.1038/srep41963

**Published:** 2017-02-06

**Authors:** Shima Momeni, Davood Nematollahi

**Affiliations:** 1 Faculty of Chemistry, Bu-Ali Sina University, Hamedan, Zip Code 65178-38683, Iran

## Abstract

Electrochemical behavior of acid orange 7 has been exhaustively studied in aqueous solutions with different pH values, using cyclic voltammetry and constant current coulometry. This study has provided new insights into the mechanistic details, pH dependence and intermediate structure of both electrochemical oxidation and reduction of acid orange 7. Surprisingly, the results indicate that a same redox couple (1-iminonaphthalen-2(1H)-one/1-aminonaphthalen-2-ol) is formed from both oxidation and reduction of acid orange 7. Also, an additional purpose of this work is electrochemical synthesis of three new derivatives of 1-amino-4-(phenylsulfonyl)naphthalen-2-ol (3a–3c) under constant current electrolysis via electrochemical oxidation (and reduction) of acid orange 7 in the presence of arylsulfinic acids as nucleophiles. The results indicate that the electrogenerated 1-iminonaphthalen-2(1 H)-one participates in Michael addition reaction with arylsulfinic acids to form the 1-amino-3-(phenylsulfonyl)naphthalen-2-ol derivatives. The synthesis was carried out in an undivided cell equipped with carbon rods as an anode and cathode.

2-Naphthol orange (acid orange 7), C_16_H_11_N_2_NaO_4_S, is a mono-azo water-soluble dye that extensively used for dyeing paper, leather and textiles^1^,^2^. The structure of acid orange 7 involves a hydroxyl group in the ortho-position to the azo group. This resulted an azo-hydrazone tautomerism, and the formation of two tautomers, which each show an acid−base equilibrium^3^,^4^,^5^,^6^,^7^,^8^,^9^,^10^,^11^,^12^. Despite the number of articles dealing with acid−base properties of the acid orange 7, this topic is not yet well known, and only one pK_a_, (pK_a_ = 11.4) is reported^6^. On the other hand, azo dyes have been widely used for developing and testing theories of color and constitution, tautomerism, indicator action, and acid-base equilibria^5^. Therefore, detailed mechanistic information is important in understanding of the stability and in identifying of the intermediates structure resulting from the oxidative or reductive decomposition of dye. Consequently, detailed mechanistic information is particularly attractive from the point of view of environmental pollution because of residual dye and the commercial applications^13^,^14^. Additionally, green/sustainable synthesis is much more important than conventional synthetic methods. The concept and significance of green sustainable chemistry (GSC), has been recognized throughout the world, and nowadays new processes cannot be developed without consideration of GSC. In recent years, much attention has been paid to electroorganic synthesis as a typical environmentally friendly process^15^,^16^,^17^,^18^,^19^,^20^,^21^,^22^,^23^. This method contains the simultaneous incidence of both oxidation (at the anode) and reduction (at the cathode). In conventional electroorganic synthesis, the synthesis of the desired products is done either by the anodic or by the cathodic reaction and so; the reaction product at the counter electrode is undesirable. The simultaneous use of both oxidation and reduction reactions to synthesis of a product is the dream of an organic electrochemist and is a wonderful strategy^24^,^25^,^26^,^27^,^28^. At ideal conditions, a 200% current efficiency is achievable for paired electrosynthesis when both anodic and cathodic reactions to provide the similar product (convergent strategy)^15^. Furthermore, sulfone compounds and naphthalene derivatives are found in antibiotic drugs such as nafacillin and 4,4-diaminodiphenylsulfone (dapsone) and antifungal drugs such as naftifine, tolnaftate and terbinafine^29^,^30^,^31^. These compounds have an effective inhibitory effect against the bacteria^30^,^31^ and antimicrobials effect against wide range of human pathogens^29^. Based on these advances, we anticipate that naphthalene derivatives containing sulfone groups reveals such properties.

The results discussed above prompted us to investigate the electrochemical oxidation and reduction of acid orange 7 in aqueous solutions with different pH values to achieve the following goals: (i) new insights into the electrochemical oxidation and reduction of acid orange 7, (ii) definitive detection of intermediates formed during the oxidative and reductive degradation of acid orange 7, and (iii) convergent paired electrochemical synthesis of new 1-amino-2-naphthol derivatives by constant current electrolysis of acid orange 7 in the presence of arylsulfinic acids as nucleophiles.

## Results and Discussion

### Electrochemical Study of Acid Orange 7

**Figure 1:**
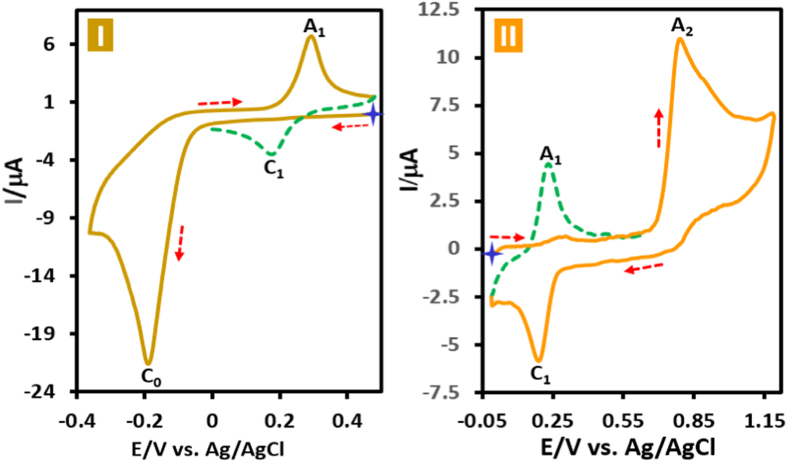
Cyclic voltammogram of **AO7** (1.0 mM) (first and second cycles) in two different direction of scanning of potential, (**I**) in negative-going scan and (**II**) in positive-going scan in aqueous phosphate buffer (*c* = 0.2 M, pH = 2.0). Scan rate: 50 mV s^−1^; T = 25 ± 1 °C.

Cyclic voltammograms of acid orange 7 (**AO7**) in aqueous phosphate buffer solution (*c* = 0.2 M, pH = 2.0) in two different potential regions (+0.5 to −0.4 and 0.0 to +1.2 V vs. Ag/AgCl) is shown in Fig. 1. When the electrode potential was scanned from +0.5 V versus Ag/AgCl to a sufficiently negative voltage (−0.4 V versus Ag/AgCl), the cyclic voltammogram exhibits a large cathodic peak (C_0_) at −0.19 with an anodic peak (A_1_) at +0.30 V versus Ag/AgCl at 50 mV s^−1^ (Fig. 1I). Under these conditions, in the second cycle a new cathodic peak (C_1_), which is the counterpart of anodic peak (A_1_) appears with an *E*_p_ value of +0.18 V versus Ag/AgCl. Moreover, when the electrode potential was scanned from 0.0 V vs. Ag/AgCl to the positive potentials (+1.2 V vs. Ag/AgCl), the voltammogram shows an anodic peak (A_2_) at +0.80 V vs. Ag/AgCl with a cathodic peak (C_1_) (Fig. 1II). As in the previous experiment, a quasi-reversible couple, A_1_/C_1_, appears in the second cycle of the voltammogram. A very important point in this study is the presence of the same redox couple in both cyclic voltammograms. This confirms the generation of the same intermediates from both oxidation and reduction of **AO7**.

When the potential scan rate increases from 250 to 8000 mV s^−1^, the cyclic voltammograms of **AO7** in anodic region (Fig. S1) show the following changes: (1) the appearance of peak C_2_, which is the counterpart of peak A_2_. (2) The increase of the anodic and cathodic peak current ratios (*I*_pA2_/*I*_pA1_) and (*I*_pC2_/*I*_pCA1_) and (3) the decrease of peak current function for anodic peak A_2_ (*I*_pA2_/v^1/2^). Increase in the potential scan rate causes a decrease in the CV time-scale and therefore a decrease in the progress of the following chemical reaction. These data confirm the occurrence of a following chemical reaction and generation of a quasi-reversible system after oxidation of **AO7**^32^. In addition, the effect of potential scan rate, *v*, (250 to 8000 mV s^−1^) on the cyclic voltammetric response of **AO7** in cathodic region which confirms the Irreversibility of the reduction process corresponding to peak C_0_ (Fig. S2).

**Figure 2:**
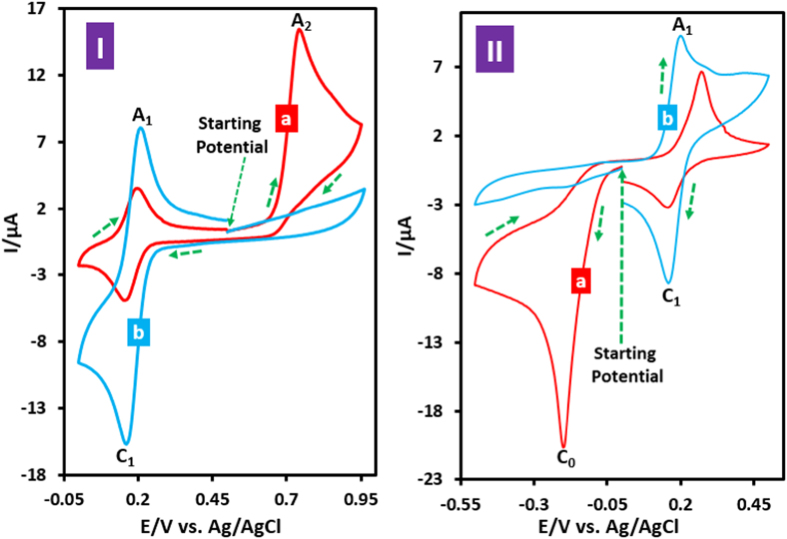
Cyclic voltammograms of **AO7** (0.1 mmol) in aqueous phosphate buffer (*c* = 0.2 M, pH = 2.0) at a glassy carbon electrode in a divided cell, during controlled potential electrolysis at: (**I**) *E*_app_ = +0.9 V and (**II**) *E*_app_ = −0.2 V vs. Ag/AgCl, (a) at the beginning of electrolysis and (b) at the end of electrolysis. Scan rate: 50 mV s^−1^. T = 25 ± 1.

**Figure 3:**
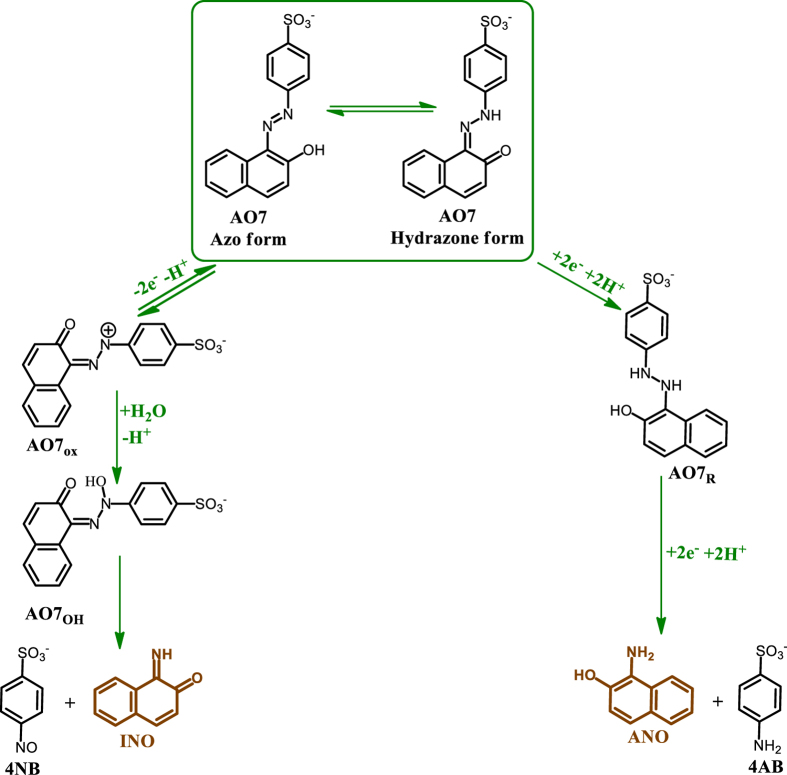
Oxidation and reduction pathways of AO7 in acidic media.

The oxidative and reductive controlled-potential coulometry of **AO7** was performed by applying potentials +0.90 and −0.20 V vs. Ag/AgCl, respectively. The solutions after coulometry are shown in Supporting Information (Fig. S3). The monitoring of the electrolysis progress was carried out by cyclic voltammetry (Fig. 2). This Figure show that, in both experiments, proportional to the advancement of coulometry, parallel to the decrease in the current of peaks A_2_ (Fig. 2I) and C_0_ (Fig. 2II), the peaks A_1_ and C_1_, increases. In these conditions, the number of transferred electrons in oxidative controlled potential coulometry was obtained 2.9 electrons pre each **AO7** molecule. On the other hand, the number of transferred electrons in reductive controlled potential coulometry was obtained 4.1 electrons pre each **AO7** molecule. An important point on the oxidation behaviour of **AO7** can be seen in Fig. 2Ia. This Figure (and also Fig. 1II) represents a typical behaviour of an *ECE* pathway in the kinetic region^32^. However, the comparison of *I*_pA2_ at the start of coulometry with *I*_pC1_ at the end of coulometry shows that, *I*_pC1_ at the end of coulometry is equal to *I*_pA2_ at the start of coulometry 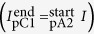. It should be noted that, in an *ECE* mechanism, the peak current ratio of the starting compound to the product (*I*_st_/*I*_pr_) in the kinetic region is {(*n*_*1*_ + *n*_*2*_*)/n*_*2*_}^3/2^. Where, *n*_1_ and *n*_2_ are the number of electrons involved in the oxidation of starting compound and product, respectively^32^. This discrepancy implying that oxidation pathway of **AO7**, is not an *ECE* and confirms the reaction pathway presented in Fig. 3, for oxidative behaviour of **AO7**. Contrary to Fig. 2I,II shows that, *I*_pC0_ at the start of coulometry, 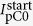, is more than 2.5 times than that of *I*_pA1_ at the end of coulometry 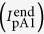 which is near to theoretical value of 2.8 when n_1_ = n_2_^33^.

Diagnostic criteria of cyclic voltammetry accompanied by previously published data on oxidation of **AO7**^34^,^35^,^36^,^37^,^38^,^39^,^40^,^41^,^42^,^43^ allow us to propose the mechanism presented in Fig. 3 for the electrochemical oxidation and reduction of **AO7**. In oxidation pathway, generation of **AO7**_**ox**_ is followed by the addition of H_2_O and formation of **AO7**_**OH**_. At the final step, **AO7**_**OH**_ degraded into 4-nitrosobenzenesulfonate (**4NB**) and 1-iminonaphthalen-2(1 *H*)-one (**INO**). It should be noted that, according to the Fig. 3, the number of transferred electron in oxidative pathway is two electrons, while, the obtained number of transferred electron (from controlled potential electrolysis) was 2.9 electrons. This discrepancy can be related to partially oxidation of **4NB**^19^.

In the reduction pathway, the first two-electron reduction converts **AO7** to **AO7**_**R**_. In the next step, **AO7**_**R**_ via an irreversible two-electron degradation process converts to 4-aminobenzenesulfonate (**4AB**) and 1-aminonaphthalen-2-ol (**ANO**).

According to the above data, the cathodic peak C_0_ corresponds to reduction of **AO7** to its reduced form (**AO7**_**R**_). The anodic peak A_2_ corresponds to the two-electron oxidation of **AO7** to its oxidized form (**AO7ox**). Obviously, the cathodic peak C_2_ corresponds to the reduction of **AO7**_**ox**_ into **AO7** and the redox couple A_1_/C_1_, are related to the oxidation of **ANO** to **INO** and reduction of **INO** to **ANO** within a quasi-reversible two-electron, two-proton process.

### Adsorption Study

Fig. S4 shows the normalized oxidative cyclic voltammograms of **AO7** (the data of Fig. S4, obtained from Fig. S1). The normalization was performed by dividing the current by the square root of the potential scan rate (*I*/v^1/2^). According to the proposed pathway for the electrochemical oxidation of **AO7**, the increasing of normalized A_2_ peak current (*I*_pA2_/v^1/2^), was unexpected. One possibility for such inconsistency is adsorption of **AO7** on the electrode surface. To confirm this finding, the plot of log *I*_PA2_ vs. log *v* at pH values 2.4, 7.2 and 10.3 is shown in Fig S5. It was reported that when the slope log *I*_PA2_ vs. log *v* is 0.5, the electrochemical reaction is a diffusion controlled process, while when the slope increases to 1, the electrochemical reaction occurs via an adsorption-controlled process^32^. It is clear that, in all pHs, the slope is more than 0.5 and increases with increasing pH from 0.58 to 0.74. These values are greater than 0.5 for the diffusion controlled process and are less than one, which is theoretical value for the adsorption-controlled electrode process. Therefore, it is clear that the electrochemical oxidation of **AO7** at glassy carbon electrode in aqueous media is adsorption/diffusion process. These results show that the interaction between anionic forms of **AO7** and the electrode surface is stronger than neutral form.

### The Effect of pH

**Figure 4:**
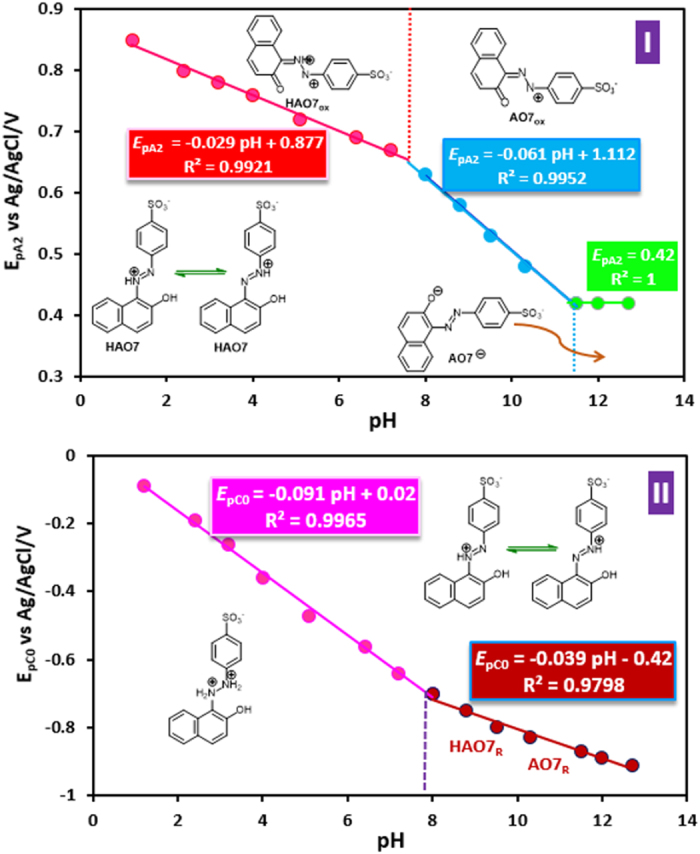
The potential-pH diagram for oxidation and reduction of AO7.

**Figure 5:**
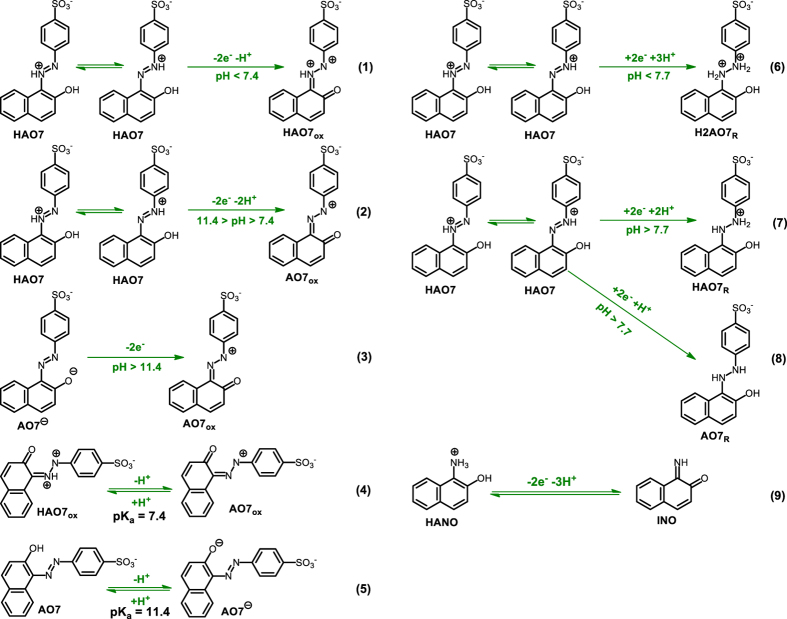
Oxidation and reduction pathways of AO7 at different pH values and acid/base equilibriums of HAO7_ox_/AO7_ox_, AO7/AO7^−^ and HANO/INO.

The electrochemical behavior of **AO7** has been studied in different pH values. The oxidative and reductive cyclic voltammograms of **AO7** in aqueous solution with various pHs are shown in Figs S6 and S7, respectively. As seen in Fig. S6, with increasing pH, *E*_pA2_ shifts to negative values. This confirms the participation of proton(s) in the oxidation of **AO7**. The variation of E_pA2_ with pH is given by:


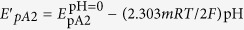


where *m* is the number of protons involved in the reaction, 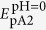
is the anodic peak potential at pH = 0.0 and *R, T*, and *F* have their usual meaning. The *E*_pA2_–pH diagram comprise three linear segments with different equations and slopes at pH values 7.4 and 11.4 (Fig. 4I). This diagram indicates that in the aqueous solutions, **AO7** is in different reduced and oxidized forms, that their relative amounts are dependent on the pH and electrode potential. At pHs lower than 7.4, the *E*_pA2_ value shifts by −29 mV/pH indicating that the redox reaction is two-electron/one-proton process involving the oxidation protonated **AO7** (**HAO7**) to protonated **AO7**_**ox**_ (Fig. 5, Eq. 1)^44^,^45^,^46^. Moreover, at pH range 7.4–11.4, the *E*_pA2_ value shifts by −61 mV/pH. In this range of pH, the redox reaction is two-electron/two-proton process involving the oxidation of **HAO7** to **AO7**_**ox**_ (Fig. 5, Eq. 2).

Finally, at pHs >11.4, the *E*_pA2_ value is independent of pH, showing that the redox reaction involves a two-electron process without participation of any proton including the oxidation of **AO7** anion (**AO7**^−^) to **AO7**_**ox**_ (Fig. 5, Eq. 3). The important point of Fig. 5 is the absence of neutral **AO7**, which can be related to the high tendency of **AO7** to keep the proton on the nitrogen atoms due to the intramolecular hydrogen bonding (Fig. S8). In addition, the calculated pK_a_ for **HAO7_ox_/AO7**_**ox**_ and **AO7**/**AO7**^−^ equilibria which is also shown in Fig. 5, Eqs 4 and 5, are: 7.4 and 11.4, respectively.

The reductive cyclic voltammograms of **AO7** in aqueous solution with different pHs are shown in Fig. S7. As seen, with increasing pH, peak C_0_ shifts to negative potentials, indicating the participation of proton(s) in the electrode process. The *E*_PC0_–pH diagram is shown in Fig. 4II. It has two linear segments at pH 7.7. At pH values lower than 7.7, *E*_pC0_ value shifts by −90 mV/pH indicating the redox reaction is two-electron/three-proton process involving the reduction of **HAO7** to **H2AO7**_**R**_ (Fig. 5, Eq. 6). However, at pHs >7.7, the situation is a little complicated, as the *E*_pC0_ value shifts by −40 mV/pH. It probably including unrecognizable reductions **HAO7** to **HAO7**_R_ (two-electron/two-proton process) and **HAO7** to **AO7**_**R**_ (two-electron/one-proton process) (Fig. 5, Eqs. 7 and 8).

The cyclic voltammograms regarding A_1_/C_1_ peaks at different pHs and its *E*_1/2_–pH diagram are shown in Fig. S9. The *E*_1/2_ values were calculated as the average of the anodic and cathodic peak potentials (*E*_pA1_ + *E*_pC1_)/2. The *E*_1/2_–pH diagram displays a simple linear dependence of the *E*_1/2_ on pH, with the slope of −85 mV/pH, indicating that the redox reaction is two-electron/three-proton process involving the oxidation of **HANO** to **INO** in the forward scan and reduction of **INO** to **HANO** in the reverse scan (Fig. 5, Eq. 9). The extrapolation of this line to pH = 0.0 provides the *E*_1/2_ = +0.60 V versus Ag/AgCl for redox couple A_1_/C_1_.

### Electrochemical Study of AO7 in the Presence of Arylsulfinic acids

**Figure 6:**
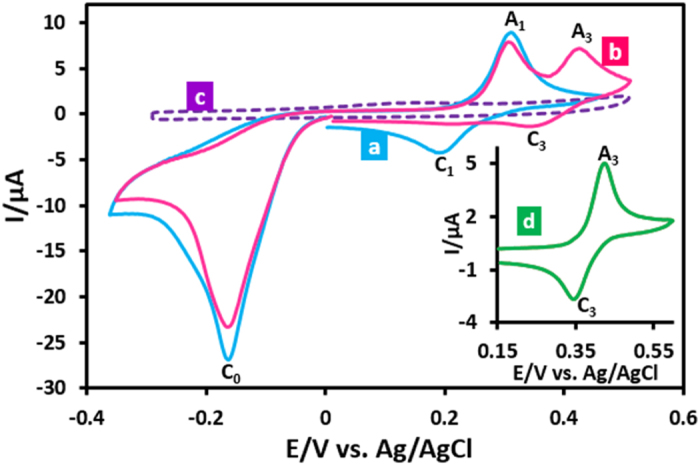
(a) Cyclic voltammogram of AO7 (1.0 mM) in the absence, (b) in the presence of 4-toluenesulfinic acid (1a) (1.0 mM), (c) 4-toluenesulfinic acid (1.0 mM) and (d) isolated product (3a) (0.05 mM), at a glassy carbon electrode, in aqueous phosphate buffer (*c* = 0.2 M, pH = 2.0). Scan rate: 100 mV s^−1^, T = 25 ± 1 °C.

**Figure 7:**
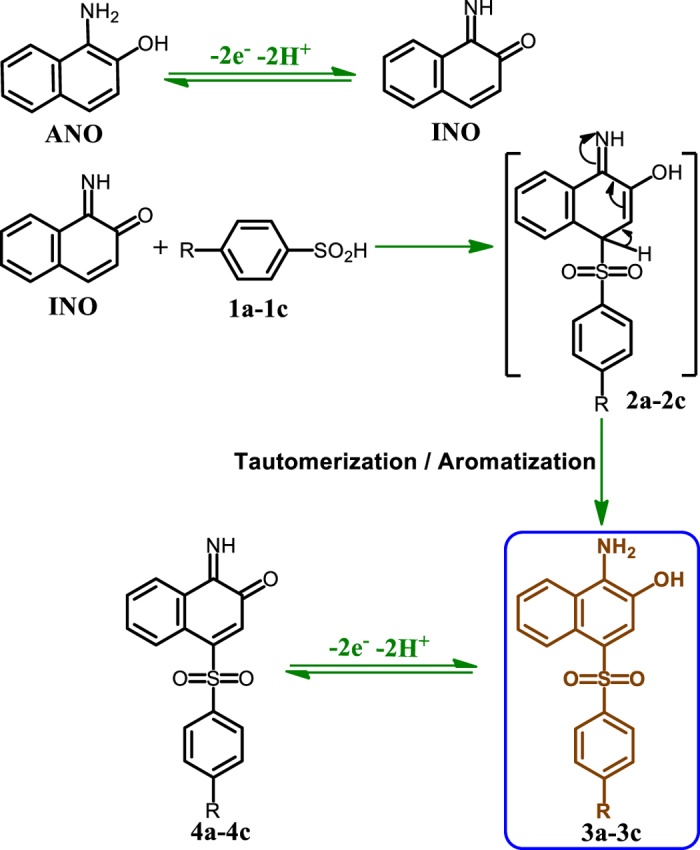
Proposed mechanism for the electrochemical oxidation of AO7 in the presence of arylsulfinic acids (1a–1c).

Because of two reasons: a) confirmation of the proposed mechanism in Fig. 3 and b) electrochemical synthesis of some new organic compounds, in this part, the electrochemical behavior of **AO7** in the presence of 4-toluenesulfinic acid (**1a**) was studied and compared with that of **AO7** in the absence of **1a** (Fig. 6). The cyclic voltammogram of **AO7** in aqueous phosphate buffer (*c* = 0.2 M, pH = 2.0), in the presence of **1a** is shown in Fig. 6b. Comparison of the voltammogram with that of **AO7** in the absence of **1a** (Fig. 6a), shows two important differences: (a) the appearance of a new redox couple (peaks A_3_ and C_3_) at *E*_1/2_ = 0.39 V vs Ag/AgCl. (b) The disappearance of the cathodic peak C_1_ in the reverse scan. Under these conditions, the peak current ratio, *I*_pC1_/*I*_pA1_, depends on the both potential scan rate and **1a** concentration, so that, *I*_pC1_/*I*_pA1_ increases with increasing scan rate and decreasing **1a** concentration. In Fig. 6, curve c is the voltammogram of **1a** in the same conditions and in the absence of **AO7** that does not show any peak in the working potential range. In addition, cyclic voltammogram d, is belong to the isolated product from the electrolysis of **AO7** in the presence of **1a**. In this part, the same results were obtained for the other two arylsulfinic acids (**1b** and **1c**). These results and the spectroscopic data of the isolated electrolysis product all point out to compound **3a** (final product), which would have been formed according to the pathway shown in Fig. 7.

**Figure 8:**
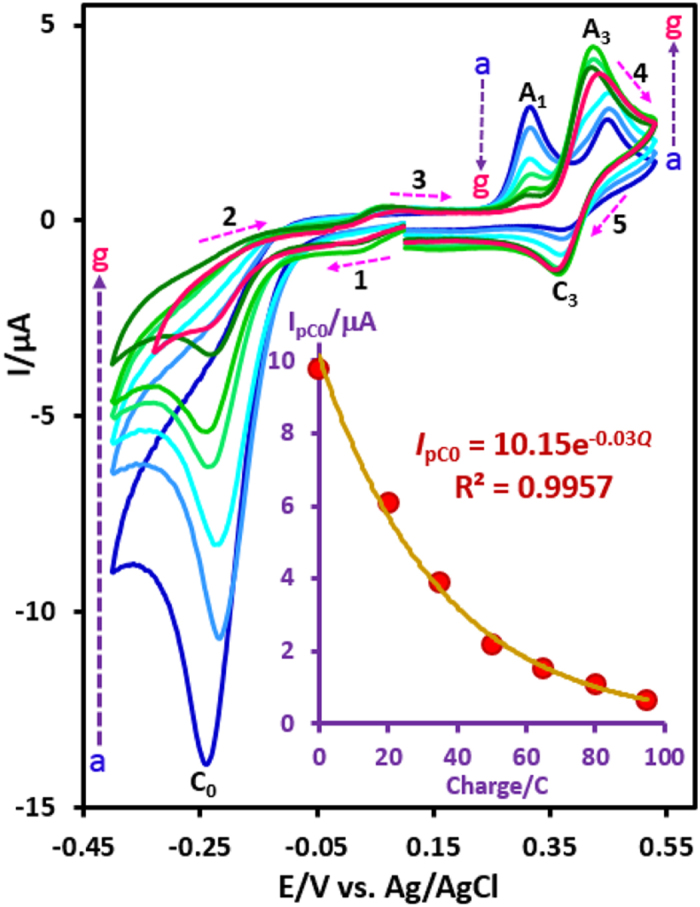
Cyclic voltammograms of AO7 (0.25 mmol) in the presence of 4-toluenesulfinic acid (1a) (0.25 mmol) in aqueous phosphate buffer (*c* = 0.2 M, pH = 2.0), at a glassy carbon electrode during constant current coulometry, after consumption of (a) 0, (b) 20, (c) 35, (d) 50, (e) 65, (f) 80 and (g) 95 C. Current density: 0.32 mA cm^−1^. Scan rate: 50 mV s^−1^. Inset: variation of peak current (*I*_pC0_) vs. charge consumed. T = 25 ± 1.

The first step in the synthesis of **3a** is the generation of **INO**. This compound is directly generated from oxidative cleavage of **AO7** (Fig. 3), and/or from the two-electron oxidation of **ANO** (Fig. 7). In the next step, **INO** would serve as a Michael acceptor in a reaction with **1a** to form the final product **3a**. The oxidation of **3a** did not occur during the electrolysis due to the insolubility of **3a** in electrolysis solution (aqueous phosphate buffer). Based on Fig. 7, the anodic and cathodic peaks A_3_ and C_3_ pertain to the oxidation of **3a** to **4a** and vice versa. According to the proposed mechanism in Fig. 3, we have designed a paired electrochemical strategy for the synthesis of the sulfone derivatives **3a–3c**. The paired electrochemical synthesis of **3a–3c** has been successfully performed in a one-pot process at the current density of 0.32 mA/cm^2^, in an undivided cell equipped with carbon rods as cathode and anode. The electrolysis was terminated when the cathodic peak that corresponds to the reduction of **AO7** (C_0_) disappears. This peak disappears after consumption of 4.0 F/mol electricity. Under these conditions, the monitoring of the electrolysis progress was carried out by cyclic voltammetry and shown in Fig. 8. This Figure shows that, proportional to the advancement of coulometry, *I*_pC0_, *I*_pA1_ and *I*_pA2_ decreases while *I*_pA3_ and *I*_pC3_ increases. The variation of *I*_pC0_ vs. charge consumed is also shown in Fig. 8 (inset). This curve show that *I*_pC0_ decrease exponentially with advancing coulometry (*I*_pC0_ = 10.15 e^−0.03*Q*^). The total amount of charge passed for terminating the reaction was determined from the extrapolation to the X-axis. The calculated charge passed confirms consumption of about 4e^−^ per molecule of **AO7**.

In addition, the UV–visible spectra of **AO7** in the presence of 4-toluenesulfinic acid (**1a**) were collected during a constant current coulometry in the same conditions as before (Fig. S11). Under these conditions, the absorption spectrum of **AO7** consists of three absorption bands at 312, 410 and 488 nm. Our data show as the coulometry is carried out, the height of all three peaks decrease and a new peak appears at 352 nm and grows in intensity.

**Figure 9:**
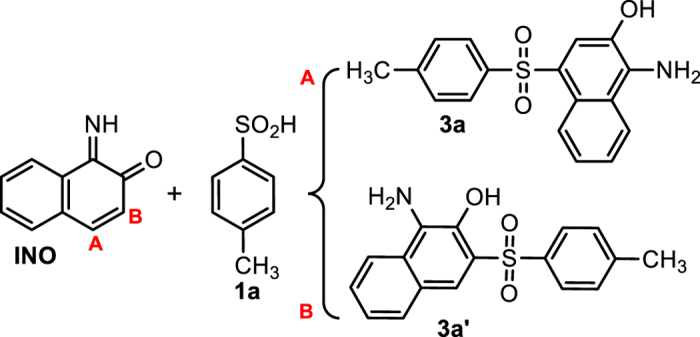
The structures of possible compounds 3a and 3a’.

**INO** is an asymmetric Michael acceptor and can be attacked by **1a** via 1,4 (site A) or 1,6 (site B) Michael addition reaction to yield two types of products (**3a** and **3a’**) (Fig. 9). However, the recorded NMR spectrum shows a singlet peak at 8.04 ppm belongs to aromatic proton. This result confirms that the 1,4-Michael addition is more probable reaction and compound **3a** is the final product of the electrochemical oxidation of **AO7** in the presence of **1a**.

In order to increase the yield of **3a–3c**, some affecting factors must be optimized. Therefore, the effects of two of the most important factors, applied current density and charge were investigated by setting all parameters to be constant and optimizing one each time. The effect of charge passed was studied in the range of 1 to 6 F mol^−1^, while the other parameters are as follows: temperature = 298 K, current density = 0.32 mA/cm^2^, electrode surface = 31.2 cm^2^, **AO7** = 0.1 mmol and **1a** = 0.1 mmol are kept constant. As is shown in Fig. S10a, the maximum product yield appears in 4.1 F mol^−1^ charge consumed. The product yield decreases with increasing charge passed from 4.1 F mol^−1^ probably due to the occurrence of side reaction(s) such as over-oxidation. Furthermore, the effect of applied current density on product yield was studied in the range 0.16–1.6 mA cm^−2^, while the other parameters (temperature = 298 K, charge consumed = 40 C, electrode surface = 31.2 cm^2^, **AO7** = 0.1 mmol, and of **1a** = 0.1 mmol) are kept constant. The results show that, with increasing the current density from 0.32 mA cm^−2^, the product yield decreases (Fig. S10b). The product yield decreasing in current densities greater than 0.32 mA cm^−2^, can be related to some side reactions such as oxidation of solvent, nucleophile or over-oxidation of **3a** and/or **INO**. To evaluate the usefulness of the pair strategy in the synthesis of **3a–3c**, electrochemical synthesis of **3a** was performed in a divided cell in both oxidative and reductive conditions. Our data confirms that in a divided cell (in both cases) (unpaired condition), (a) the yield of **3a** is lower and (b) the charge consumption is greater than that of in undivided cell.

## Conclusions

This work provides new insights into the electrochemical behavior of **AO7** in aqueous solutions in both oxidative and reductive regions and shows that both oxidation and reduction of **AO7** leading to the formation of a redox couple (**ANO**/**INO**) (Fig. 3). In addition, the pH dependence of **AO7** and other intermediates was studied in order to understand the predominant species, oxidation and reduction pathways and adsorption study. For example, our data shows that, the interaction between anionic forms of **AO7** and the electrode surface is stronger than neutral form. Furthermore, in this work, the electrochemical oxidation/reduction of **AO7** has been investigated in the presence of arylsulfinic acids (**1a–1c**) as nucleophiles, in acidic solutions. Our data display that the intermediate (**INO**) is attacked by the nucleophile, **1a–1c**, to give the final product **3a–3c** (Fig. 7). Clean synthesis, technical feasibility (using galvanostatic method and simple cell), use of electricity instead of oxidative or reductive reagents, one-step process, work in room temperature and pressure and use of aqueous solution instead of organic solvents, are the advantages of this method.

## Materials and Methods

### Apparatus and Reagents

Cyclic voltammetry, controlled-potential coulometry and preparative electrolysis were performed using an Autolab model PGSTAT 30 and a Behpazho potentiostat/galvanostat. The working and counter electrode used in macro-scale electrolysis and coulometry was an assembly of four ordinary soft carbon rods (6 mm diameter and 4 cm length). Working electrode used in the cyclic voltammetry experiments was a glassy carbon disc (1.8 mm diameter) and a platinum rod was used as a counter electrode. The electrosynthesis were performed under constant-current condition in an undivided cell. The glassy carbon electrode was polished using alumina slurry (from Iran Alumina Co.). More details are described in our previous paper^47^. Acid orange 7, arylsulfinic acids and phosphate salts were obtained from commercial sources. These chemicals were used without further purification.

### Electroorganic Synthesis of 3a–3c

An aqueous phosphate buffer solution (70 ml, *c* = 0.2 M, pH 2.0) containing **AO7** (0.25 mmol) and arylsulfinic acid (**1a–1c**) (0.25 mmol) was electrolyzed in an undivided cell under constant current conditions (current density = 0.32 mA cm^−2^) for 2 h 45 min. At the end of electrolysis, the cell was placed in a refrigerator overnight. The precipitated solid was collected by filtration and washed several times with water. After recrystallization in ethyl ether, the products were characterized by IR, ^1^H NMR, ^13^C NMR and mass spectroscopy.

### 1-Amino-3-tosylnaphthalen-2-ol (C_17_H_15_NO_3_S) (3a)

mp: 163–164 °C; isolated yield 65%. ^1^H NMR (400 MHz, DMSO-*d*_6_): *δ* = 2.32 (s, 3 H, methyl), 6.22 (s, ~1 H, NH, this peak disappeared upon addition of D_2_O), 7.36 (m, 4 H, *J* = 8 Hz, aromatic), 7.71 (d, 2 H, *J* = 8.4, aromatic), 8.04 (s, 1 H, aromatic), 8.11 (m, 1 H, aromatic), 8.29 (m, 1 H, aromatic), 9.86 (s, ~1 H, OH, this peak disappeared upon addition of D_2_O); ^13^C NMR (100 MHz, DMSO-*d*_6_): *δ* = 21.3, 119.5, 120.5, 122.7, 123.1, 123.9, 124.8, 125.0, 125.8, 126.9, 130.2, 136.7, 137.7, 140.4, 143.7; IR (KBr): 3384, 2926, 1704, 1622, 1354, 1266, 1200, 1140, 1083, 951, 755, 668, 571, 529 cm^−1^; MS (EI, 70 eV): m/z (relative intensity %): 313 (M^+^, 31), 270 (6), 158 (100), 139 (11), 130 (65), 91 (24), 77 (15), 65(18).

### 1-amino-3-(phenylsulfonyl)naphthalen-2-ol (C_16_H_13_NO_3_S) (3b)

mp: 209–210 °C; isolated yield 60%. ^1^H NMR (400 MHz, DMSO-*d*_6_): *δ* = 6.27 (s, ~1 H, NH, this peak disappeared upon addition of D_2_O), 7.38 (dd, 2 H, *J* = 3.2 and 10.0 Hz, aromatic), 7.57 (m, 3 H, aromatic), 7.85 (dd, 2 H, *J* = 2.0 and 6.8 Hz, aromatic), 8.09 (s, 1 H, aromatic), 8.18 (dd, 1 H, *J* = 3.2 and 10.0 Hz, aromatic), 8.33 (dd, 1 H, *J* = 3.2 and 10.0 Hz, aromatic), 9.91 (s, ~1 H, OH, this peak disappeared upon addition of D_2_O); ^13^C NMR (100 MHz, DMSO-*d*_6_): *δ* = 118.8, 120.7, 122.7, 123.2, 123.9, 124.7, 125.1, 125.8, 126.8, 129.8, 133.1, 136.8, 138.2, 143.4; IR (KBr): 3473, 3379, 3073, 3025, 1739, 1616, 1358, 1286, 1207, 1136, 1082, 952, 784, 740, 557 cm^−1^; MS (EI, 70 eV): m/z (relative intensity %): 299 (M^+^, 21), 257 (7), 160 (16), 159 (55), 158 (100), 130 (58), 103 (16), 77(44), 51(24), 43 (74).

### 1-amino-3-((4-chlorophenyl)sulfonyl)naphthalen-2-ol (C_16_H_12_ClNO_3_S) (3c)

mp: 164–165 °C; isolated yield 62%. ^1^H NMR (400 MHz, DMSO-*d*_6_): *δ* = 6.33 (s, ~1 H, NH, this peak disappeared upon addition of D_2_O), 7.38 (dd, 2 H, *J* = 3.2 and 9.6 Hz, aromatic), 7.62 (d, 2 H, *J* = 8.8 Hz, aromatic), 7.85 (d, 2 H, *J* = 8.8 Hz, aromatic), 8.06 (d, 1 H, *J* = 2.8 Hz, aromatic), 8.19 (m, 1 H, aromatic), 8.28 (m, 1 H, aromatic), 9.92 (s, ~1 H, OH, this peak disappeared upon addition of D_2_O); ^13^C NMR (100 MHz, DMSO-*d*_6_): *δ* = 118.2, 120.6, 122.6, 123.1, 123.7, 124.3, 124.9, 125.0, 126.2, 128.8, 129.9, 136.7, 138.2, 142.0; IR (KBr): 3362, 3286, 3068, 1630, 1309, 1284, 1174, 1144, 1085, 906, 824, 781, 752, 648, 580 cm^−1^; MS (EI, 70 eV): m/z (relative intensity %): 333 (M^+^, 24), 174 (12), 158 (100), 130 (58), 111 (21), 103 (18), 77 (17), 75 (28), 50 (18).

## Additional Information

**How to cite this article:** Momeni, S. and Nematollahi, D. New insights into the electrochemical behavior of acid orange 7: Convergent paired electrochemical synthesis of new aminonaphthol derivatives. *Sci. Rep.* **7**, 41963; doi: 10.1038/srep41963 (2017).

**Publisher's note:** Springer Nature remains neutral with regard to jurisdictional claims in published maps and institutional affiliations.

## Supplementary Material

Supporting Information

## References

[b1] Silva J. P. . Adsorption of acid orange 7 dye in aqueous solutions by spent brewery grains. Sep. Purif. Technol. 40, 309–315 (2004).

[b2] Zollinger H. Color Chemistry: Syntheses, Properties, and Applications of Organic Dyes and Pigments. (John Wiley & Sons, 2003).

[b3] Hihara T., Okada Y. & Morita Z. Azo-hydrazone tautomerism of phenylazonaphthol sulfonates and their analysis using the semiempirical molecular orbital PM5 method. Dyes Pigm. 59, 25–41 (2003).

[b4] Özen A. S., Doruker P. & Aviyente V. Effect of cooperative hydrogen bonding in azo-hydrazone tautomerism of azo dyes. J. Phys. Chem. A 111, 13506–13514 (2007). 18052263 10.1021/jp0755645

[b5] Gordon P. F. & Gregory P. Organic Chemistry in Colour (Springer Science & Business Media, 2012).

[b6] Oakes J. & Gratton P. Kinetic investigations of azo dye oxidation in aqueous media. J. Chem. Soc. Perk. T. 2, 1857–1864 (1998).

[b7] Oakes J., Gratton P., Clark R. & Wilkes I. Kinetic investigation of the oxidation of substituted arylazonaphthol dyes by hydrogen peroxide in alkaline solution. J. Chem. Soc. Perk. T. 2, 2569–2576 (1998).

[b8] Hihara T., Okada Y. & Morita Z. Reactivity of phenylazonaphthol sulfonates, their estimation by semiempirical molecular orbital PM5 method, and the relation between their reactivity and azo-hydrazone tautomerism. Dyes Pigm. 59, 201–222 (2003).

[b9] Allen R. L. Colour Chemistry (Springer Science & Business Media, 2013).

[b10] Christie R. Colour Chemistry (Royal Society of Chemistry, 2014).

[b11] Thomas F. & Boto K. The Chemistry of the Hydrazo, Azo and Azoxy Groups. 443–493 (Wiley-VCH, Weinheim, 1975).

[b12] Ember E., Rothbart S., Puchta R. & van Eldik R. Metal ion-catalyzed oxidative degradation of orange II by H_2_O_2_. High catalytic activity of simple manganese salts. New J. Chem. 33, 34–49 (2009).

[b13] Robinson T., McMullan G., Marchant R. & Nigam P. Remediation of dyes in textile effluent: a critical review on current treatment technologies with a proposed alternative. Bioresource Technol. 77, 247–255 (2001). 10.1016/s0960-8524(00)00080-8 11272011

[b14] Waring D. R. & Hallas G. The Chemistry and Application of Dyes (Springer Science & Business Media, 2013).

[b15] Fuchigami T., Atobe M. & Inagi S. Fundamentals and Applications of Organic Electrochemistry: Synthesis, Materials, Devices (John Wiley & Sons, 2014).

[b16] Varmaghani F., Nematollahi D., Mallakpour S. & Esmaili R. Electrochemical oxidation of 4-substituted urazoles in the presence of arylsulfinic acids: an efficient method for the synthesis of new sulfonamide derivatives. Green Chem. 14, 963–967 (2012).

[b17] Salehzadeh H., Nematollahi D. & Hesari H. An efficient electrochemical method for the atom economical synthesis of some benzoxazole derivatives. Green Chem. 15, 2441–2446 (2013).

[b18] Nematollahi D., Hosseiny Davarani S. S. & Mirahmadpour P. A green approach for the electroorganic synthesis of new dihydroxyphenyl-indolin-2-one derivatives. ACS Sustainable Chem. Eng. 2, 579–583 (2014).

[b19] Khazalpour S. & Nematollahi D. Electrochemical and chemical synthesis of different types of sulfonamide derivatives of *N,N*-dimethyl-1,4-benzenediamine using 4-nitroso-*Nα*-dimethylaniline. Green Chem. 17, 3508–3514 (2015).

[b20] Maleki A., Nematollahi D., Rasouli F. & Zeinodini-Meimand A. Electrode instead of catalyst and enzyme. A greener protocol for the synthesis of new 2-hydroxyacetamide derivatives containing a γ-lactone ring. Green Chem. 18, 672–675 (2016).

[b21] Nematollahi D. & Rafiee M. Diversity in electrochemical oxidation of dihydroxybenzoic acids in the presence of acetylacetone. A green method for synthesis of new benzofuran derivatives. Green Chem. 7, 638–644 (2005).

[b22] Salahifar E., Nematollahi D., Bayat M., Mahyari A. & Amiri Rudbari H. Regioselective green electrochemical approach to the synthesis of nitroacetaminophen derivatives. Org. Lett. 17, 4666–4669 (2015). 26381590 10.1021/acs.orglett.5b01837

[b23] Paddon C. A., Pritchard G. J., Thiemann T. & Marken F. Paired electrosynthesis: micro-flow cell processes with and without added electrolyte. Electrochem. Commun. 4, 825–831 (2002).

[b24] Amatore C., Lund H. & Baizer M. Organic Electrochemistry (Marcel Dekker, New York, 1991).

[b25] Hammerich O. & Lund H. Organic Electrochemistry (CRC Press, 2000).

[b26] Nematollahi D. & Varmaghani F. Paired electrochemical synthesis of new organosulfone derivatives. Electrochim. Acta 53, 3350–3355 (2008)

[b27] Habibi D., Pakravan N. & Nematollahi D. The green and convergent paired Diels–Alder electro-synthetic reaction of 1,4-hydroquinone with 1,2-bis (bromomethyl) benzene. Electrochem. Commun. 49, 65–69 (2014).

[b28] Sharafi-Kolkeshvandi M., Nematollahi D. & Nikpour F. A Green CC bond formation reaction between *N,N*′-diphenylbenzene-1, 4-diamine and Michael donors: A convergent paired strategy. J. Electrochem. Soc. 163, G75–G78 (2016).

[b29] Beale J. M. Jr. & Block J. (eds) Wilson and Gisvold’s Textbook of Organic Medicinal and Pharmaceutical Chemistry. 12th ed. (Lippincott Williams and Wilkins, Philadelphia, 2011).

[b30] Nematollahi D., Baniardalan M., Khazalpour S. & Pajohi-Alamoti M. R. Product diversity by changing the electrode potential. Synthesis, kinetic evaluation and antibacterial activity of arylsulfonyl-4,4′-biphenol and bis-arylsulfonyl-4, 4′-biphenol derivatives. Electrochim. Acta 191, 98–105 (2016).

[b31] Khazalpour S., Nematollahi D. & Pajohi-Alamoti M. R. A green approach for the synthesis of bis (substituted sulfabenzamide) *para*-benzoquinone based on the reaction of sulfabenzamide with electrochemically generated *para*-benzoquinone and its antibacterial evaluation. New J. Chem. 39, 6734–6737 (2015).

[b32] Bard A. J. & Faulkner L. R. Electrochemical Methods: Fundamentals and Applications (Wiley, 2000).

[b33] Nicholson R. S. & Shain I. Theory of stationary electrode polarography. Single scan and cyclic methods applied to reversible, irreversible, and kinetic systems. Anal. Chem. 36, 706–723 (1964).

[b34] Abbott L. C., Batchelor S. N., Smith J. R. L. & Moore J. N. Reductive reaction mechanisms of the azo dye orange II in aqueous solution and in cellulose: from radical intermediates to products. J. Phys. Chem. A 113, 6091–6103 (2009). 19413355 10.1021/jp9021147

[b35] Mu Y., Rabaey K., Rozendal R. A., Yuan Z. & Keller J. Decolorization of azo dyes in bioelectrochemical systems. Environ. Sci. Technol. 43, 5137–5143 (2009). 19673319 10.1021/es900057f

[b36] Zhang S. J., Yu H. Q. & Li Q. R. Radiolytic degradation of acid orange 7: A mechanistic study. Chemosphere 61, 1003–1011 (2005). 15885740 10.1016/j.chemosphere.2005.03.008

[b37] Lopez C. . Mechanism of enzymatic degradation of the azo dye orange II determined by *ex situ*^1^H nuclear magnetic resonance and electrospray ionization-ion trap mass spectrometry. Anal. Biochem. 335, 135–149 (2004). 15519581 10.1016/j.ab.2004.08.037

[b38] Ozcan A., Oturan M. A., Oturan N. & Şahin Y. Removal of acid orange 7 from water by electrochemically generated Fenton’s reagent. J. Hazard. Mater. 163, 1213–1220 (2009). 18804327 10.1016/j.jhazmat.2008.07.088

[b39] Hammami S., Bellakhal N., Oturan N., Oturan M. A. & Dachraoui M. Degradation of acid orange 7 by electrochemically generated OH radicals in acidic aqueous medium using a boron-doped diamond or platinum anode: A mechanistic study. Chemosphere 73, 678–684 (2008).; 18760822 10.1016/j.chemosphere.2008.07.010

[b40] Wu J., Zhang H. & Qiu J. Degradation of acid orange 7 in aqueous solution by a novel electro/Fe ^2+^/peroxydisulfate process. J. Hazard. Mater. 215, 138–145 (2012). 22421343 10.1016/j.jhazmat.2012.02.047

[b41] Zhao H. Z., Sun Y., Xu L. N. & Ni J. R. Removal of acid orange 7 in simulated wastewater using a three-dimensional electrode reactor: Removal mechanisms and dye degradation pathway. Chemosphere 78, 46–51 (2010). 19897229 10.1016/j.chemosphere.2009.10.034

[b42] Zheng J., Gao Z., He H., Yang S. & Sun C. Efficient degradation of acid orange 7 in aqueous solution by iron ore tailing Fenton-like process. Chemosphere 150, 40–48 (2016). 26891355 10.1016/j.chemosphere.2016.02.001

[b43] Cai C., Zhang Z. & Zhang H. Electro-assisted heterogeneous activation of persulfate by Fe/SBA-15 for the degradation of orange II. J. Hazard. Mater. 313, 209–218 (2016). 27124213 10.1016/j.jhazmat.2016.04.007

[b44] Baymaky M. S. & Zuman P. Equilibria of formation and dehydration of the carbinolamine intermediate in the reaction of benzaldehyde with hydrazine. Tetrahedron 63, 5450–5454 (2007).

[b45] Baymak M. S., Celik H., Ludvik J., Lund H. & Zuman P. Diprotonated hydrazones and oximes as reactive intermediates in electrochemical reductions. Tetrahedron Lett. 45, 5113–5115 (2004).

[b46] Shine H. J., Zmuda H. & Park K. H. Mechanism of the benzidine rearrangement. Kinetic isotope effects and transition states. Evidence for concerted rearrangement. J. Am. Chem. Soc. 103, 955–956 (1981).

[b47] Nematollahi D., Dehdashtian S. & Niazi A. Electrochemical oxidation of some dihydroxybenzene derivatives in the presence of indole. J. Electroanal. Chem. 616, 79–86 (2008).

