# Supplementary material for: New insights into the electrochemical behavior of acid orange 7: Convergent paired electrochemical synthesis of new aminonaphthol derivatives
Source: Sci Rep. 2017 Feb 6;7:41963. doi: 10.1038/srep41963 (PMC5292738; doi:10.1038/srep41963)

Supplementary Information for

New insights into the electrochemical behavior of acid orange 7 in aqueous solutions: Convergent paired electrochemical synthesis of new aminonaphthol derivatives

Shima Momeni-Azandariani, Davood Nematollahi*

Faculty of Chemistry, Bu-Ali Sina University, Hamedan, Zip Code 65178-38683.

Iran

Table of Contents:

**1**	Cyclic voltammograms of **AO7** (anodic direction) (Fig. S1)……………….	**Page 1**
**2**	Cyclic voltammograms of **AO7** (cathodic direction) (Fig. S2)……………..	**Page 2**
**3**	Solutions after controlled-potential coulometry of **AO7** (Fig. S3)…………	**Page 3**
**4**	Normalized cyclic voltammograms of **AO7** (Fig. S4)……………………...	**Page 4**
**5**	The plots of log I_{pA2} vs. log v (Fig. S5)……………………………………..	**Page 5**
**6**	Cyclic voltammograms of **AO7** in positive-going scan (Fig. S6)…………..	**Page 6**
**7**	Cyclic voltammograms of **AO7** in negative-going scan (Fig. S7)…………	**Page 7**
**8**	Possible intramolecular hydrogen bonding (Fig. S8)………………………	**Page 8**
**9**	Cyclic voltammograms of **AO7** in positive-going scan (Fig. S9)…………..	**Page 9**
**10**	The effect of charge passed and current density (Fig. S10)………………...	**Page 10**
**11**	Absorption spectra of **AO7** in the presence of **1a** during CCC……………..	**Page 11**
**12**	Cyclic voltammograms and absorption spectra of **3a** and its oxidized form	**Page 12**
**11**	IR spectrum of **3a**…………………………………………………………...	**Page 13**
**12**	^{1}H NMR spectrum of **3a**…………………………………………………	**Page 14**
**13**	^{1}H NMR spectrum of **3a** (with D_2O)………………………………………..	**Page 15**
**14**	Expanded ^{1}H NMR spectrum of **3a**…………………………………….......	**Page 16**
**15**	^{13}C NMR spectrum of **3a**…………………………………………………	**Page 17**
**16**	MS spectrum of **3a**…………………………………………………………..	**Page 18**
**17**	IR spectrum of **3b**…………………………………………………………...	**Page 19**
**18**	^{1}H NMR spectrum of **3b**…………………………………………………	**Page 20**
**19**	Expanded ^{1}H NMR spectrum of **3b**………………………………………	**Page 21**

20 ^{13}C NMR spectrum of **3b**………………………………………………………… **Page 22**

21 MS spectrum of **3b**…………………………………………………………… **Page 23**

22 IR spectrum of **3c**……………………………………………………………… **Page 24**

23 ^{1}H NMR spectrum of **3c**………………………………………………………. **Page 25**

24 ^{1}H NMR spectrum of **3c** (with D_2O)……………………………………….. **Page 26**

25 Expanded ^{1}H NMR spectrum of **3c**……………………………………………. **Page 27**

26 ^{13}C NMR spectrum of **3c**………………………………………………………… **Page 28**

27 MS spectrum of **3c**…………………………………………………………….. **Page 29**

- **Cyclic voltammograms of AO7 (anodic direction)**

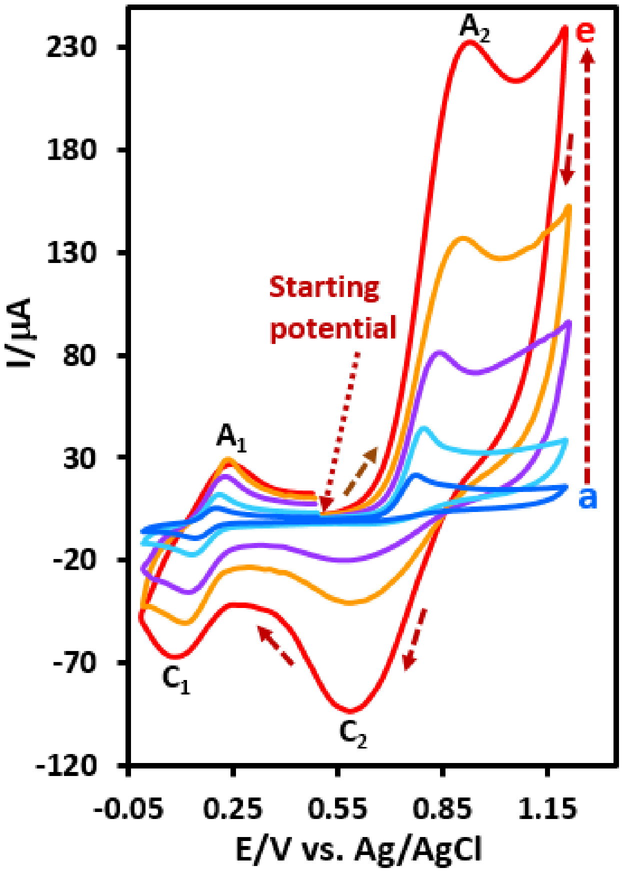

Figure S1. Cyclic voltammograms of **AO7** (1.0 mM) at a glassy carbon electrode, in aqueous phosphate buffer (*c* = 0.2 M, pH = 2.0) at various potential scan rates. Scan rates from (a) to (e) are: 0.25, 1, 3, 5 and 8 V s^{-1}, respectively. T = 25 ± 1◦C.

- **Cyclic voltammograms of AO7 (cathodic direction)**

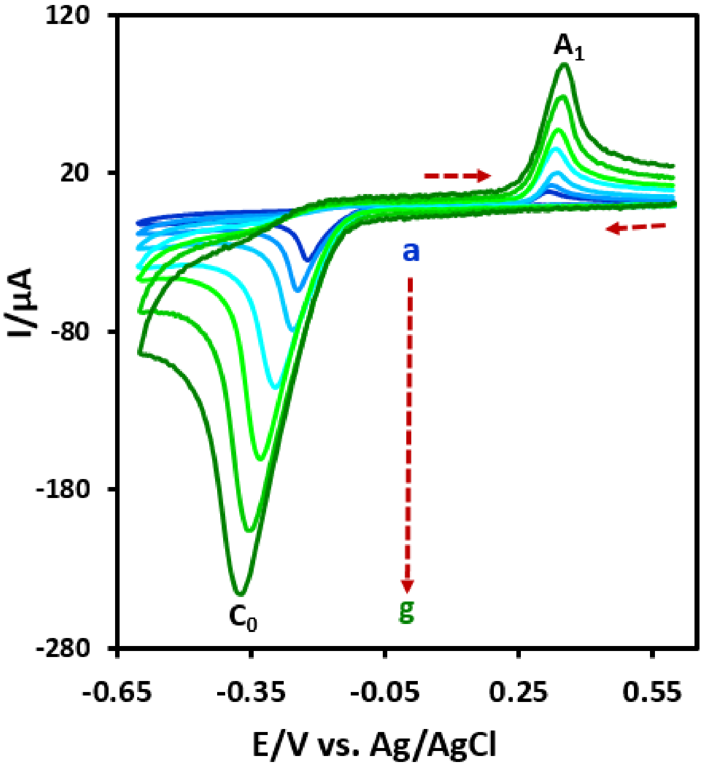

Figure S2. Cyclic voltammograms of **AO7** (1.0 mM) at a glassy carbon electrode, in aqueous phosphate buffer (c = 0.2 M, pH = 2.0) at different potential scan rates. Scan rates from (a) to (g) are: 0.25, 0.5, 1, 2, 4, 6 and 8 V s^{-1}, respectively. T = 25 ± 1 ◦C.

- **Solutions after controlled-potential coulometry of AO7**

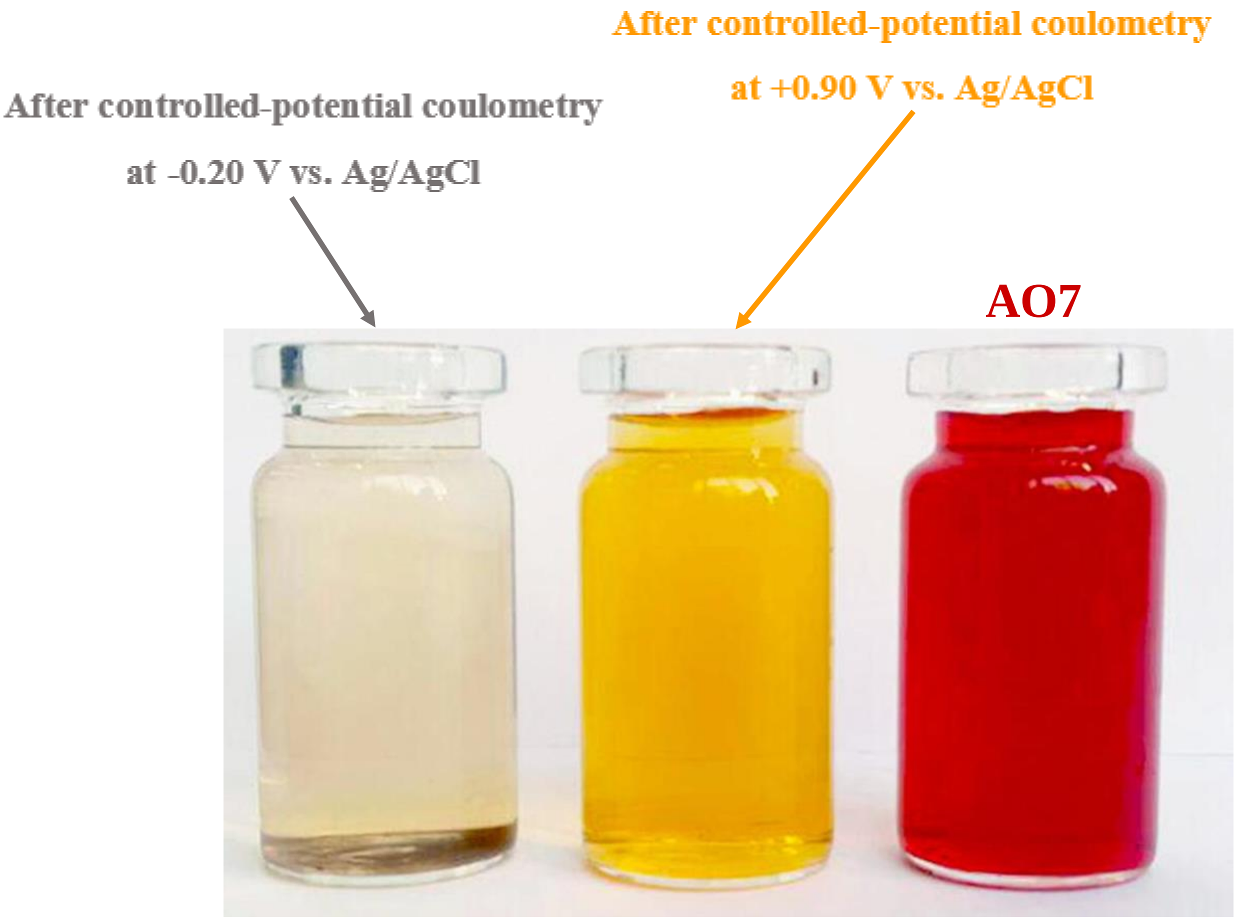

Figure S3. Solutions after controlled-potential coulometry of **AO7**

- **Normalized cyclic voltammograms of AO7**

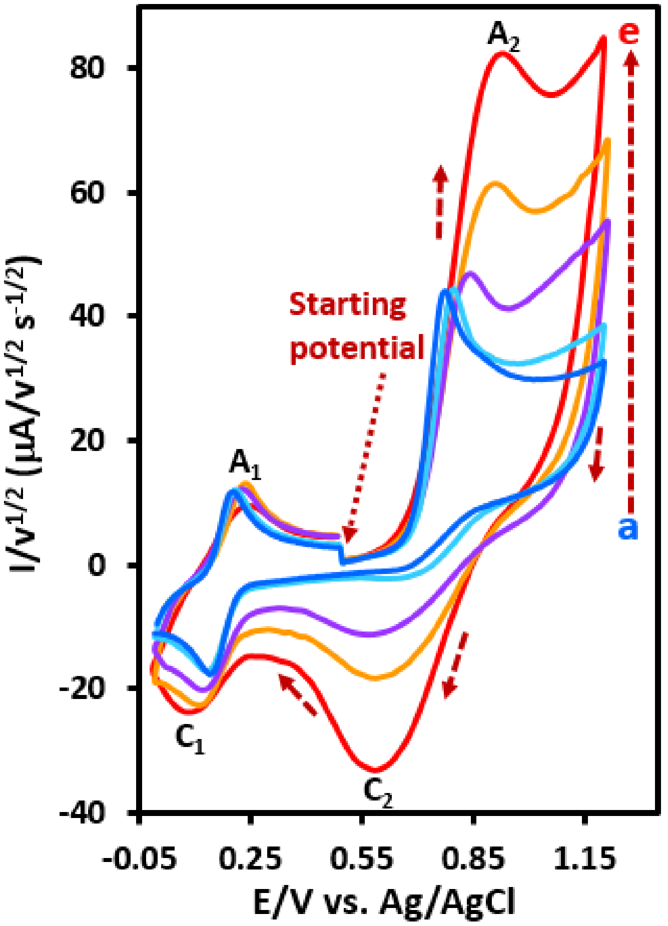

Figure S4. Normalized cyclic voltammograms of Fig S1. T = 25 ± 1◦C.

- **The plots of log I_{pA2} vs. log v**

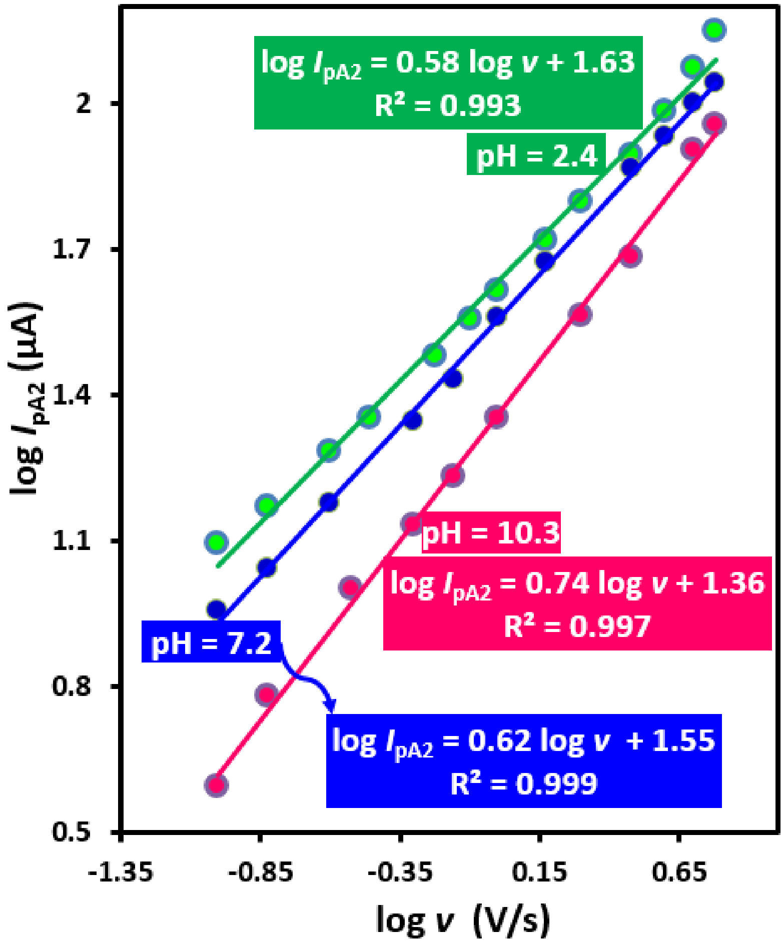

Figure S5. The plots of log I_{pA2} vs. log v at different pH values.

- **Cyclic voltammograms of AO7 in positive-going scan**

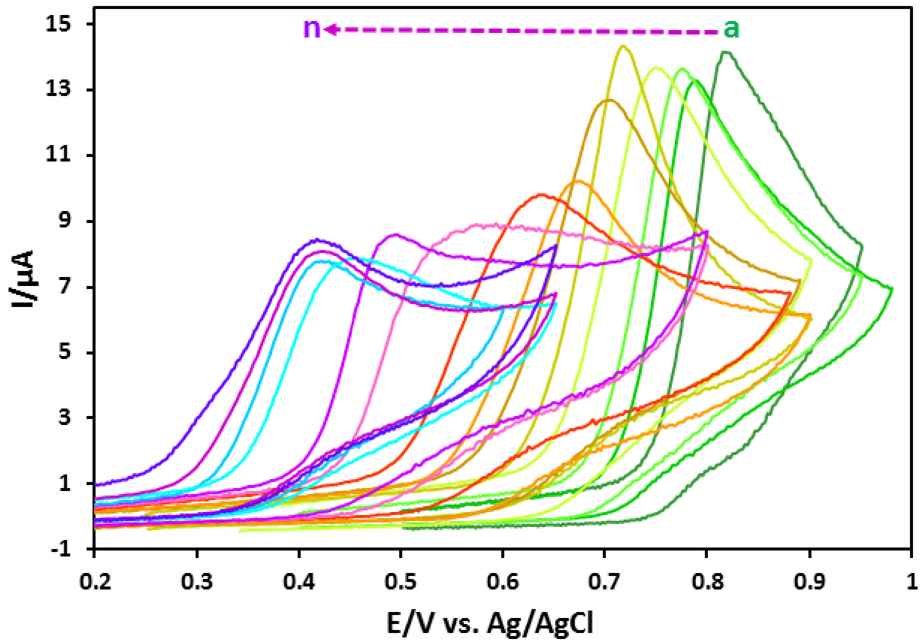

Figure S6. Cyclic voltammograms of **AO7** (1.0 mM) in positive-going scan at a glassy carbon electrode, in aqueous phosphate buffer (c = 0.2 M, pH = 2.0) with different pH values and same ionic strength. pHs from (a) to (n) are: 1.2, 2.4, 3.2, 4.0, 5.1, 6.4, 7.2, 8, 8.8, 9.5, 10.3, 11.5, 12 and 12.7. Scan rate 50 mV s^{-1}. T = 25 ± 1◦C.

- ## Cyclic voltammograms of AO7 in negative-going scan

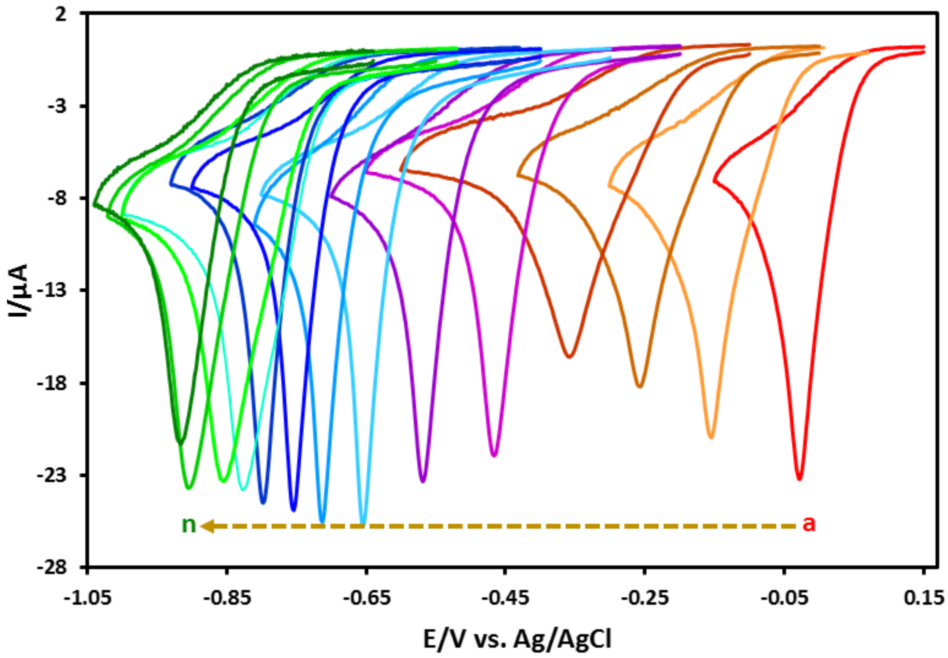

Figure S7. Cyclic voltammograms of **AO7** (1.0 mM) in negative-going scan at a glassy carbon electrode, in aqueous phosphate buffer (c = 0.2 M, pH = 2.0) with different pH values and same ionic strength. pHs from (a) to (n) are: 1.2, 2.4, 3.2, 4.0, 5.1, 6.4, 7.2, 8, 8.8, 9.5, 10.3, 11.5, 12 and 12.7. Scan rate 50 mV s^{-1}. T = 25 ± 1◦C.

- **Possible intramolecular hydrogen bonding**

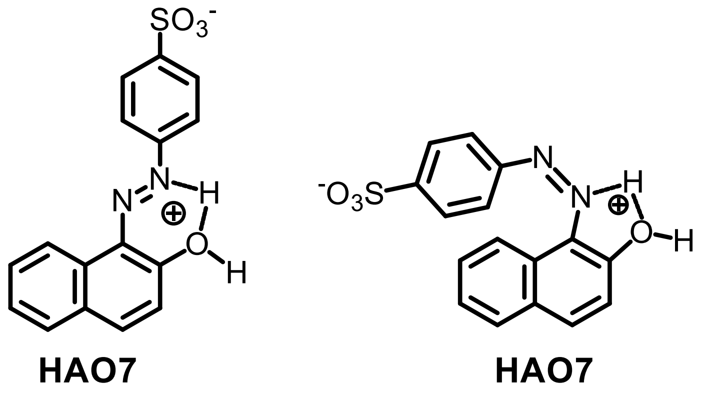

Figure S8. Possible intramolecular hydrogen bonding of protonated **AO7** (**HAO7**).

- ## Cyclic voltammograms of AO7 in positive-going scan

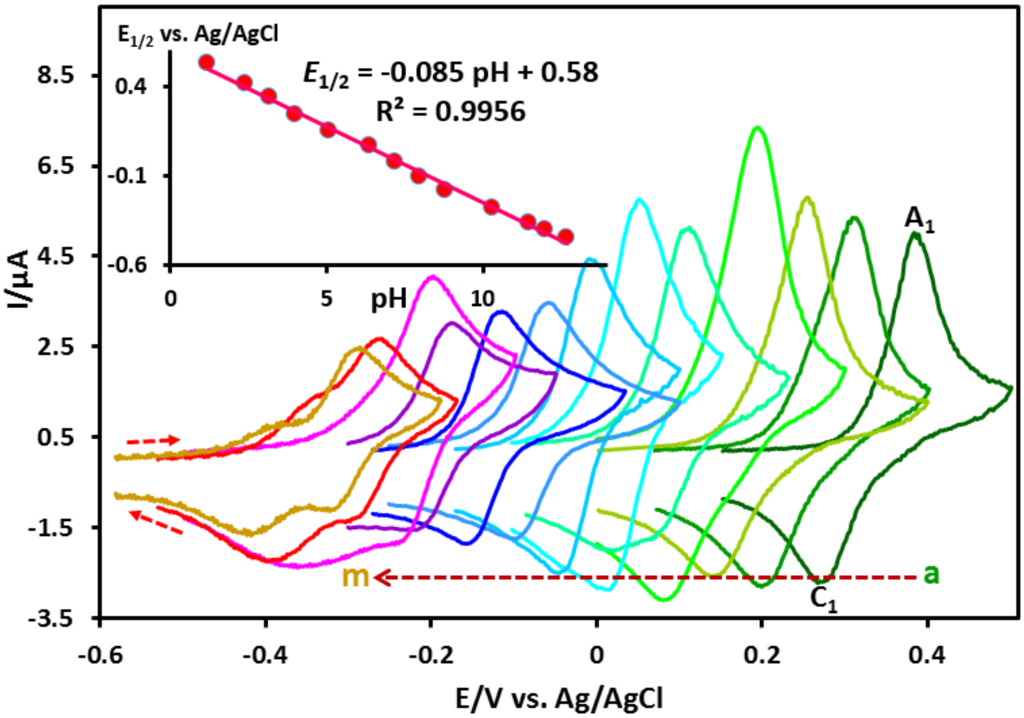

Figure S9. Cyclic voltammograms of **AO7** (1.0 mM) in positive-going scan at a glassy carbon electrode, in aqueous phosphate buffer (c = 0.2 M, pH = 2.0) with different pH values and same ionic strength. pHs from (a) to (n) are: 1.2, 2.4, 3.2, 4.0, 5.1, 6.4, 7.2, 8, 8.8, 10.3, 11.5, 12 and 12.7. Scan rate 50 mV s^{-1}. T = 25 ± 1◦C.

- **The effect of charge passed and current density**

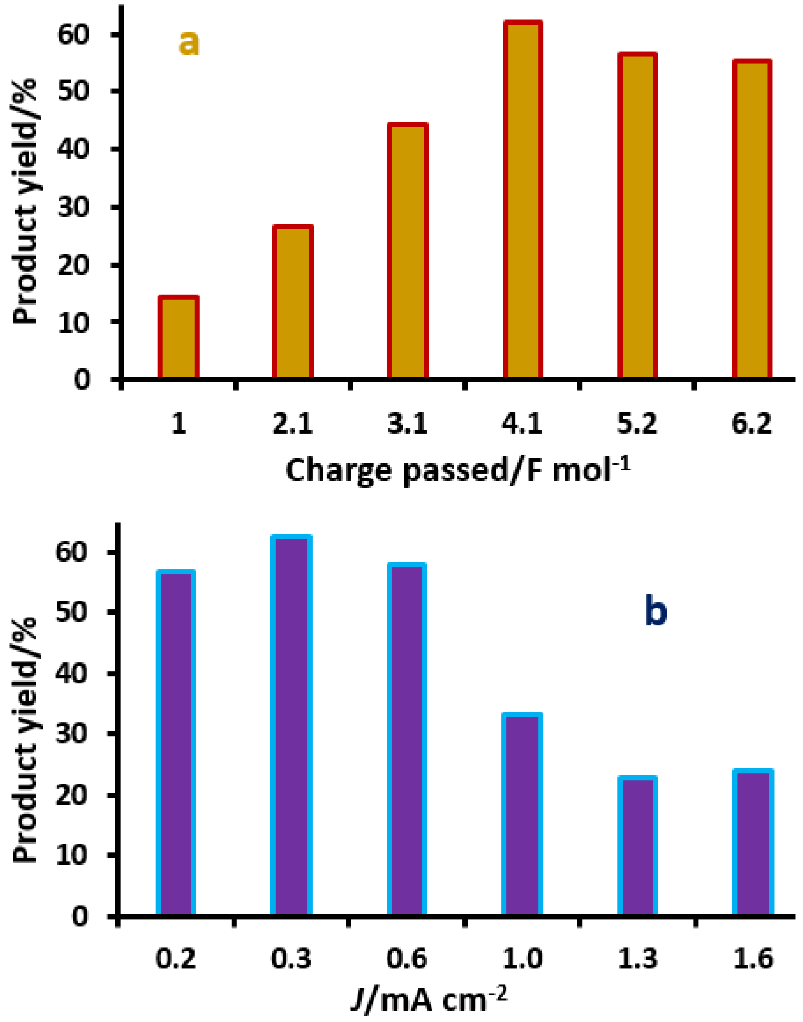

Figure S10. (a) The effect of charge passed and (b) current density on the product yield.

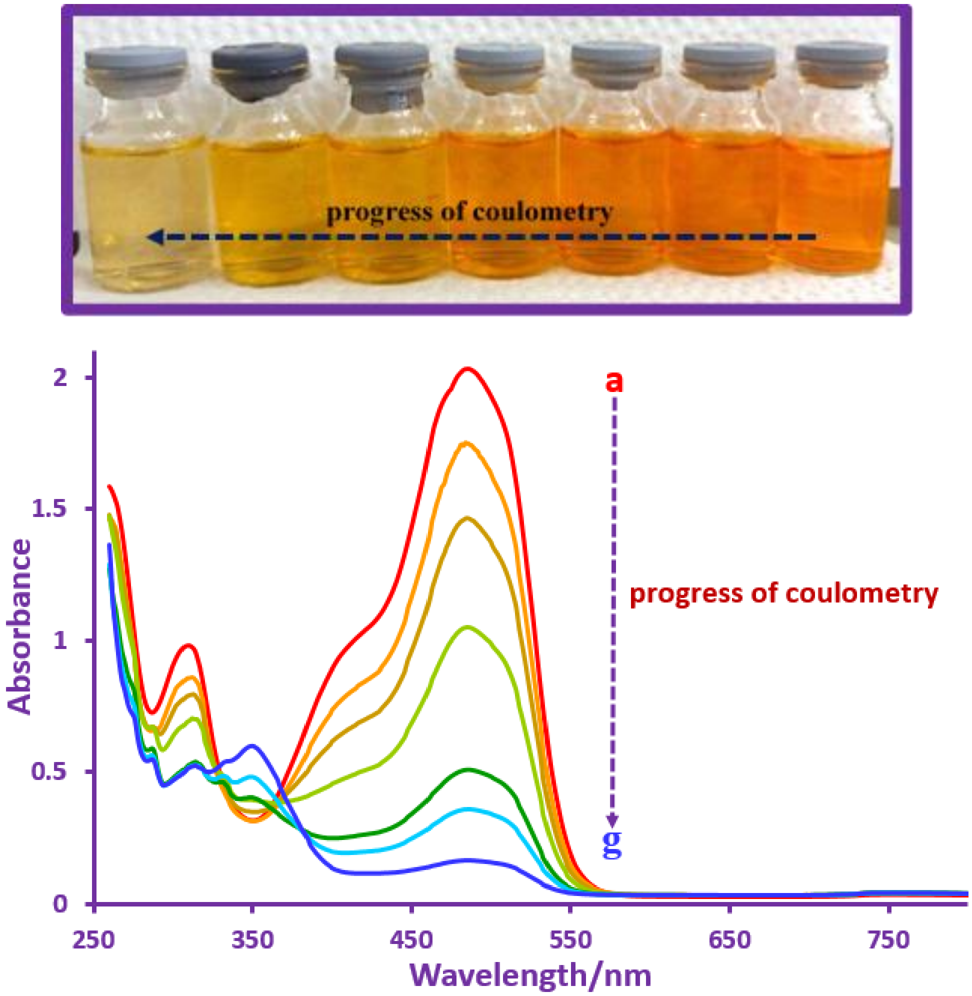

Figure S11. Solution samples and absorption spectra of **AO7** (0.25 mmol) in the presence of 4-toluenesulfinic acid (**1a**) (0.25 mmol) in aqueous phosphate buffer (c = 0.2 M, pH = 2.0), during constant current coulometry, after consumption of (a) 0, (b) 15, (c) 30, (d) 45, (e) 60, (f) 75 and (g) 90 C. Applied current density: 0.32 mA cm^{-1}.

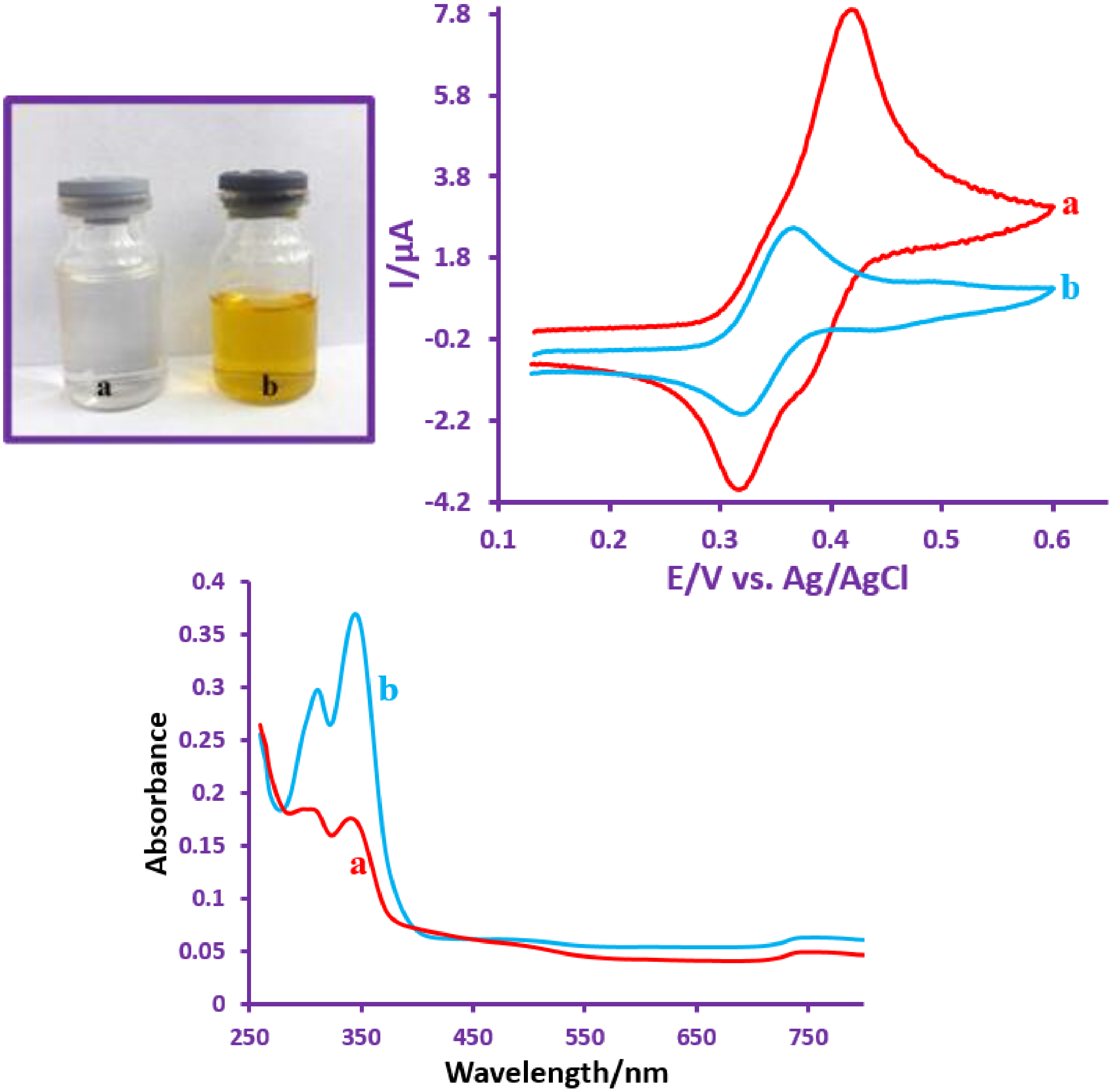

Figure S12. Solution samles, cyclic voltammograms and absorption spectra of **3a** (0.02 mmol) and its oxidized form (by constant current coulometry) (b) in water (phosphate buffer, c = 0.2 M, pH = 2.0)/ethanol mixture (50/50, v/v). (a) at the beginning of electrolysis and (b) at the end of electrolysis. Applied current density: 0.32 mA cm^{-1}.

- **IR spectrum of 3a**

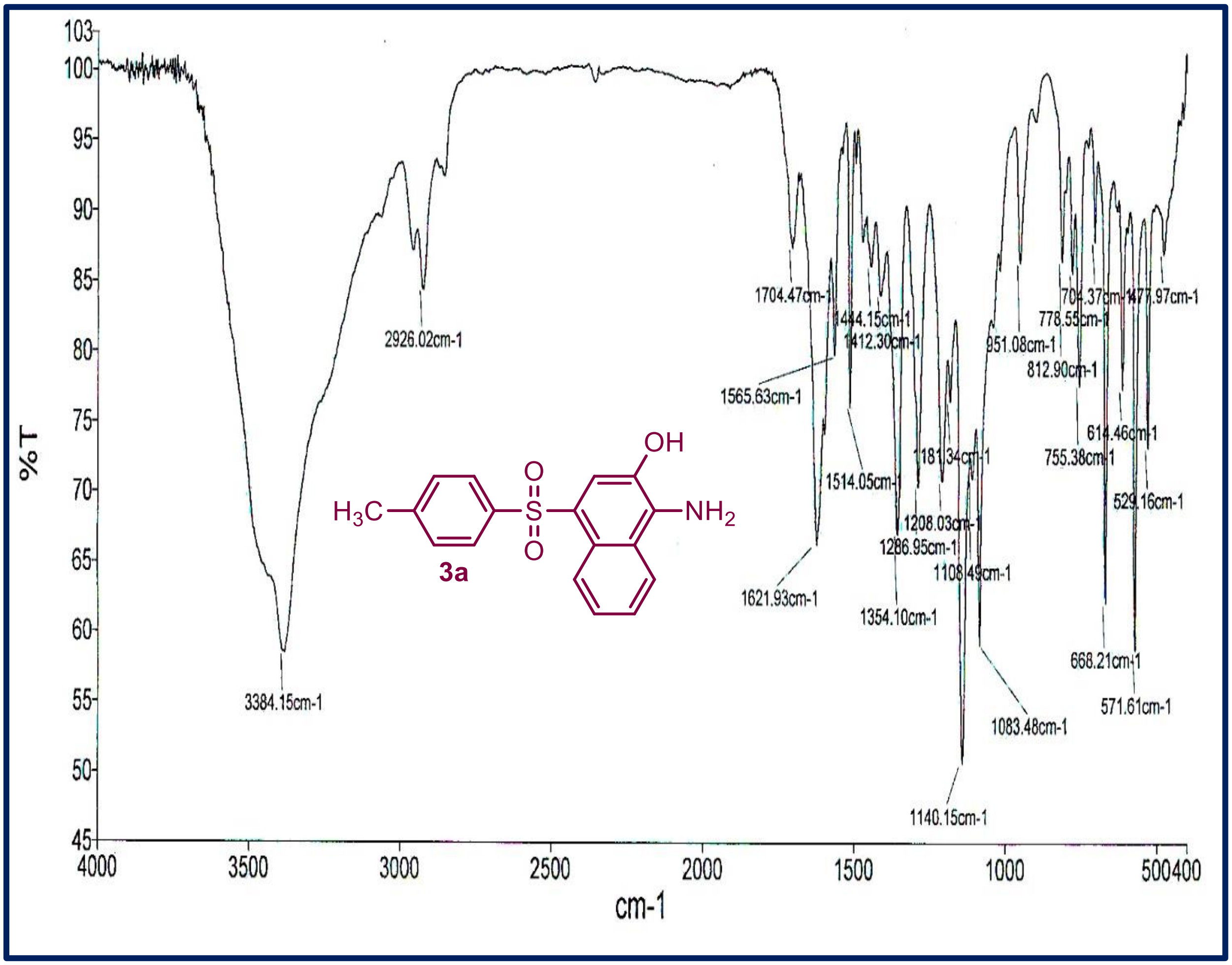

- ^{1}H NMR spectrum of 3a

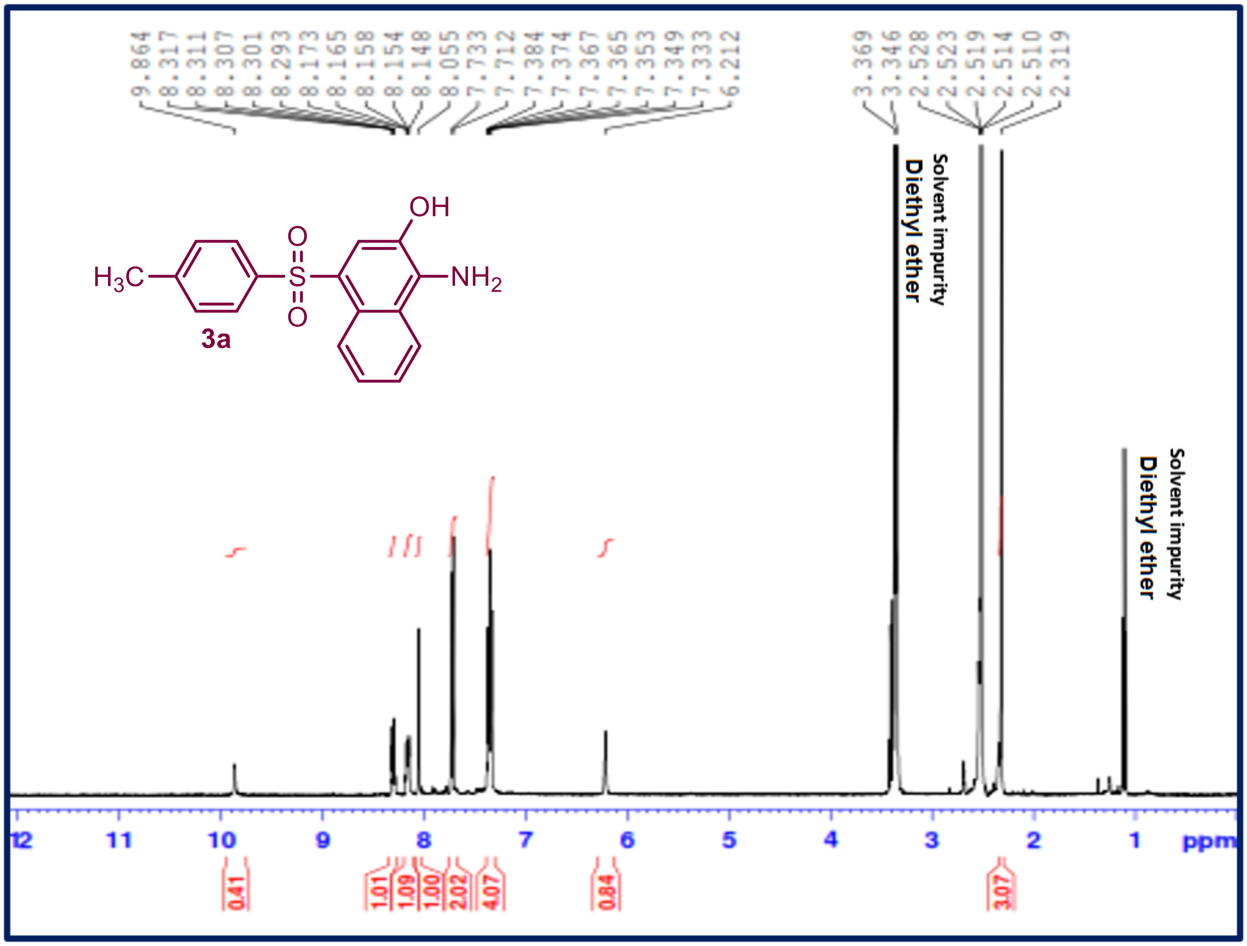

- ^{1}H NMR spectrum of 3a in the presence of D_2O

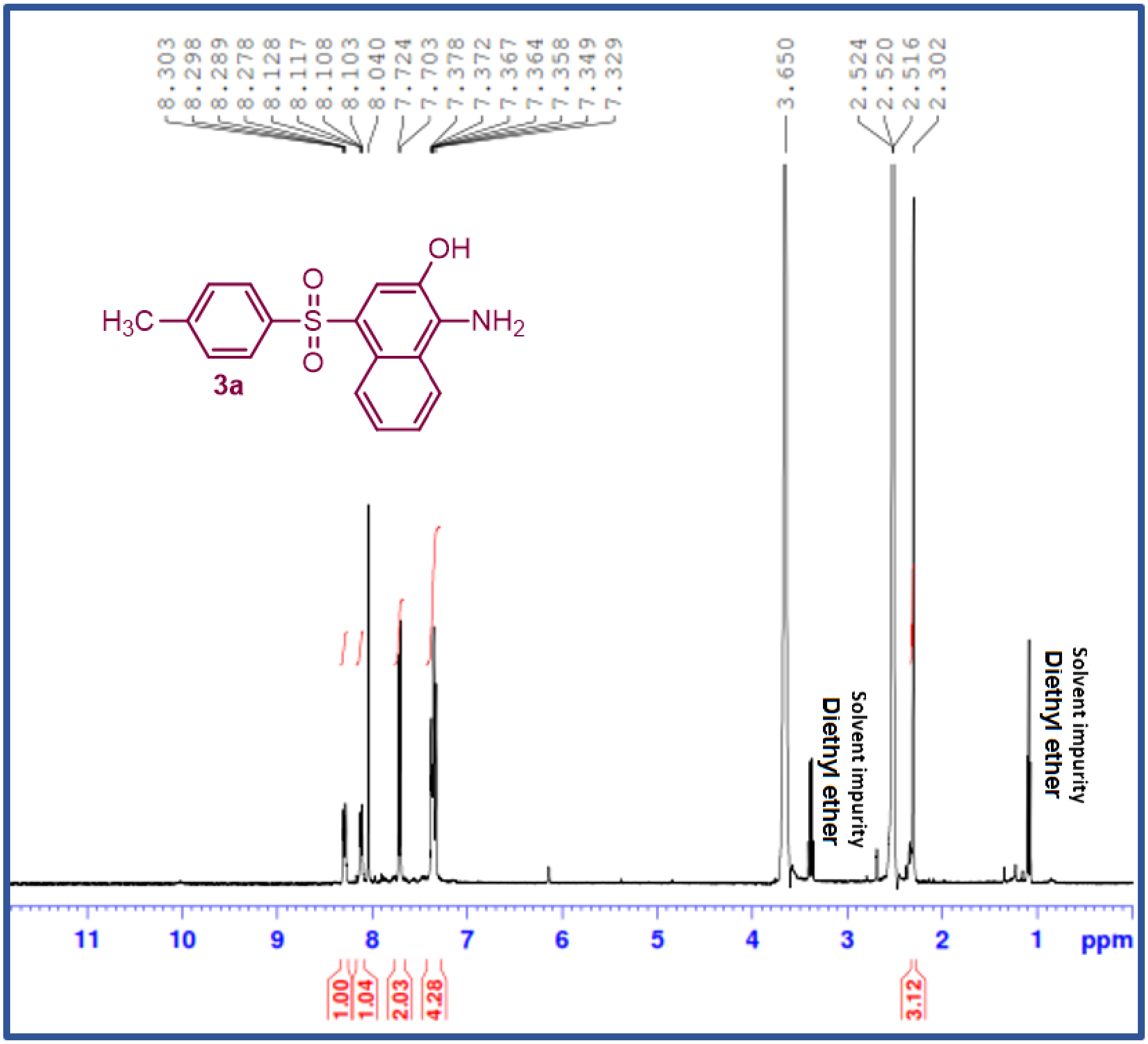

- **Expanded ^{1}H NMR spectrum of 3a**

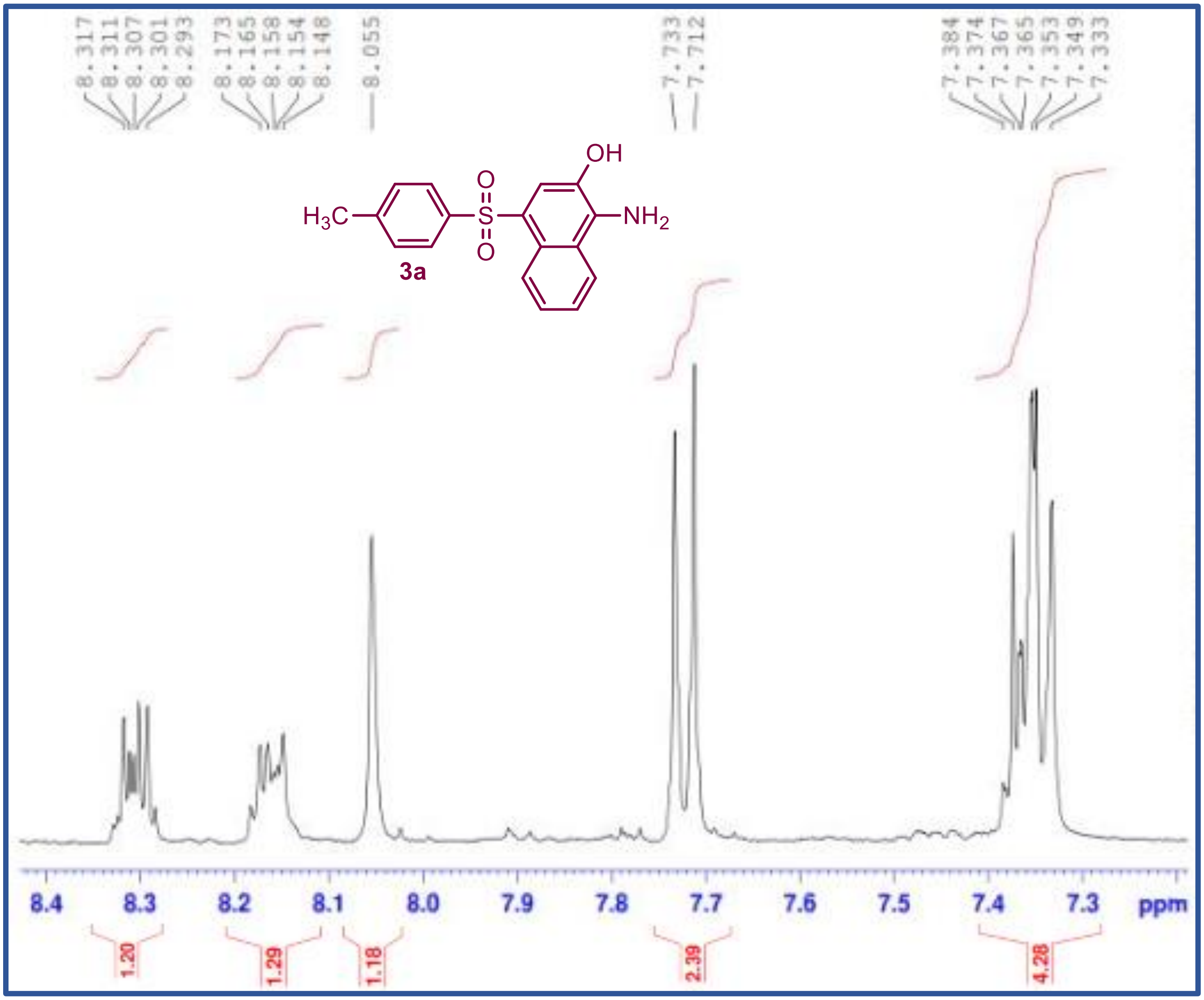

• ^{13}C NMR spectrum of 3a

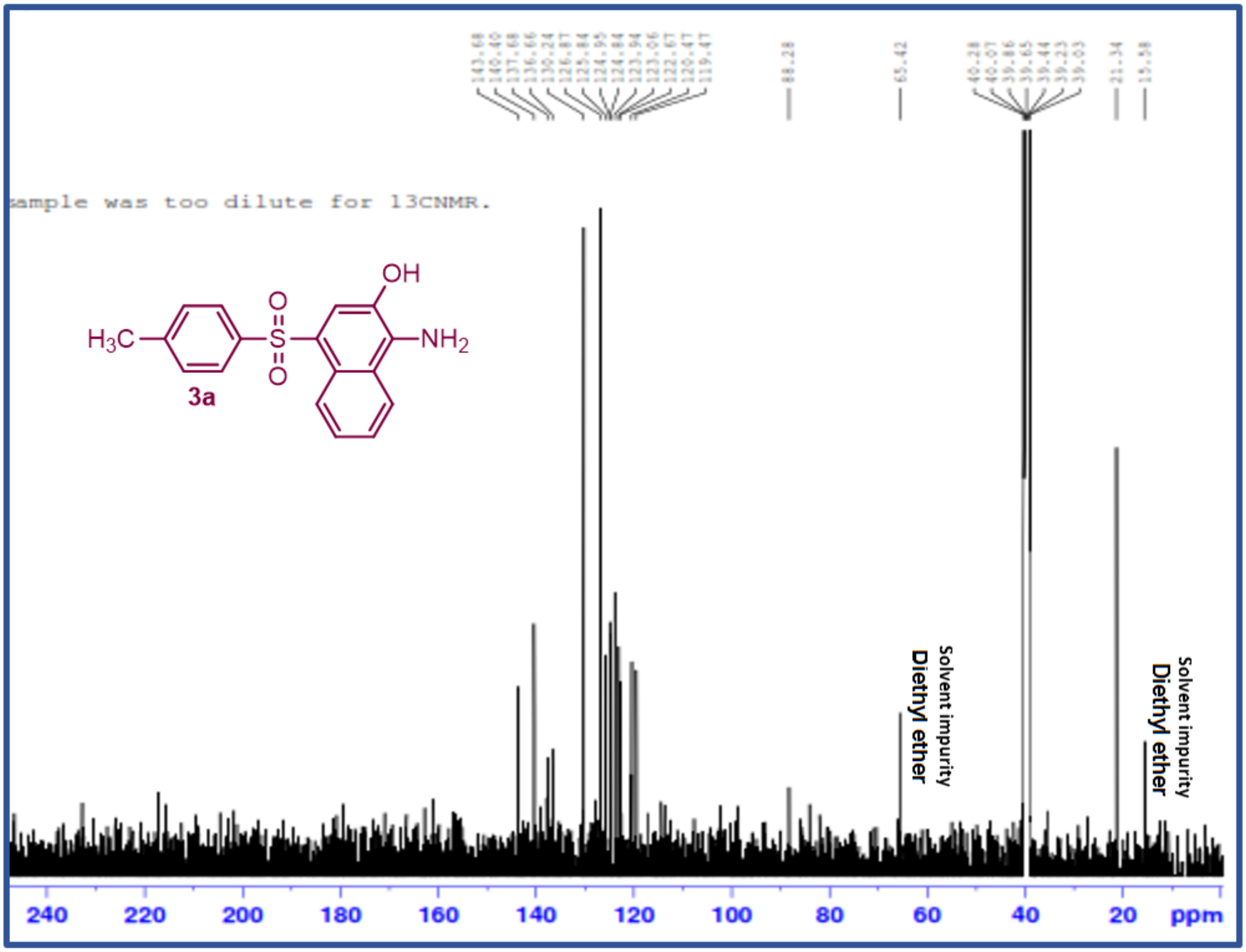

- **MS spectrum of 3a**

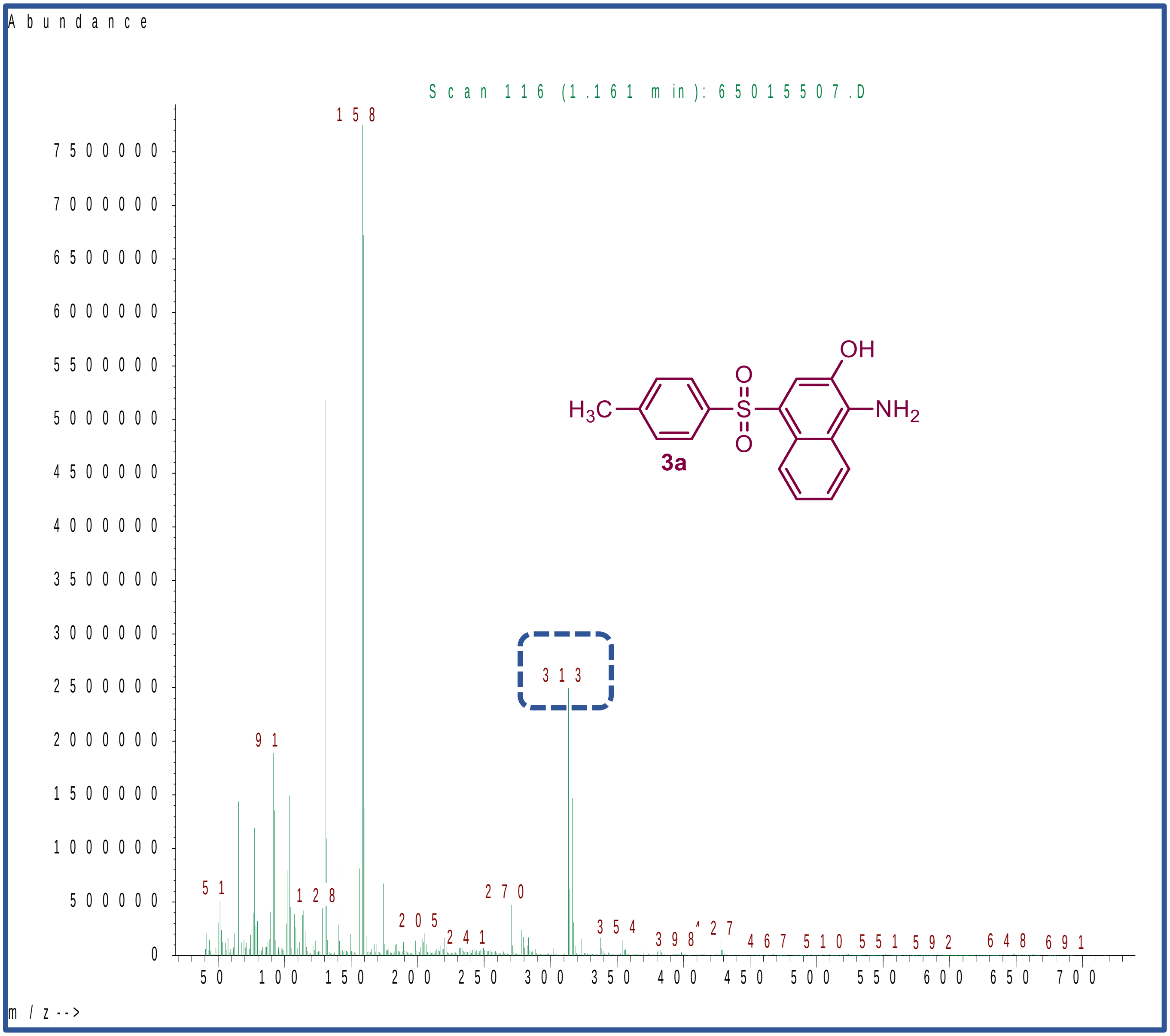

- **IR spectrum of 3b**

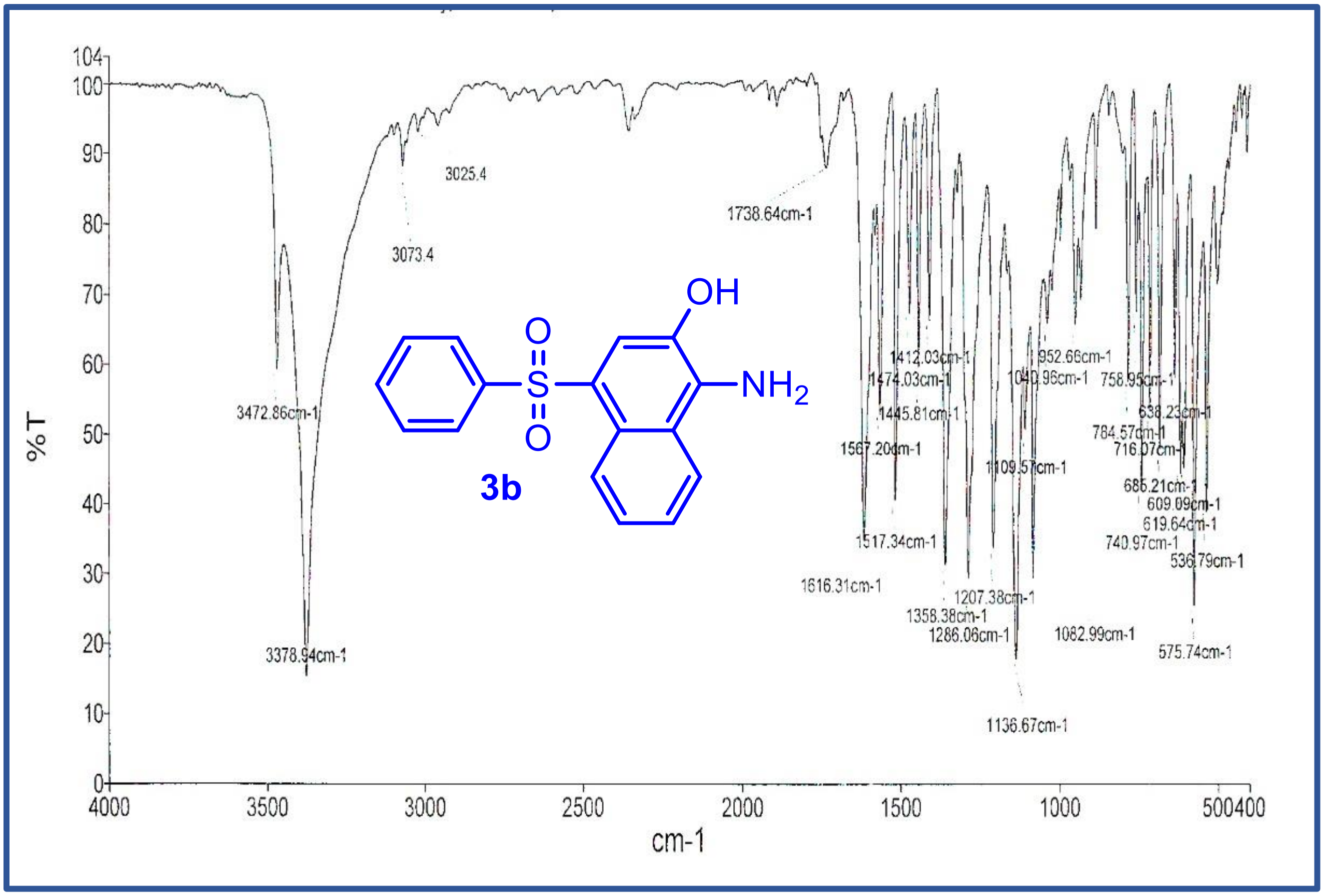

- ^{1}H NMR spectrum of 3b

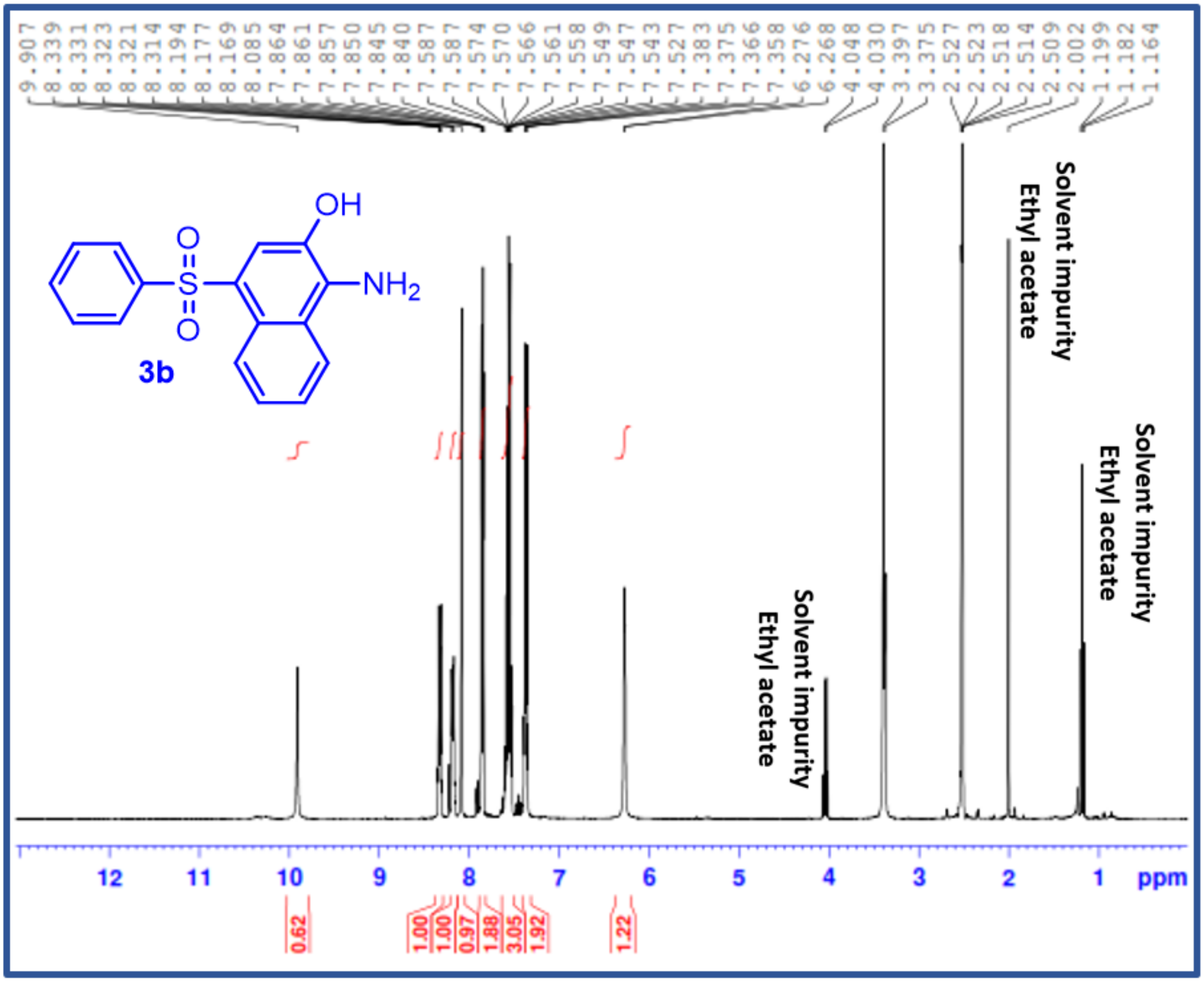

- **Expanded ^{1}H NMR spectrum of 3b**

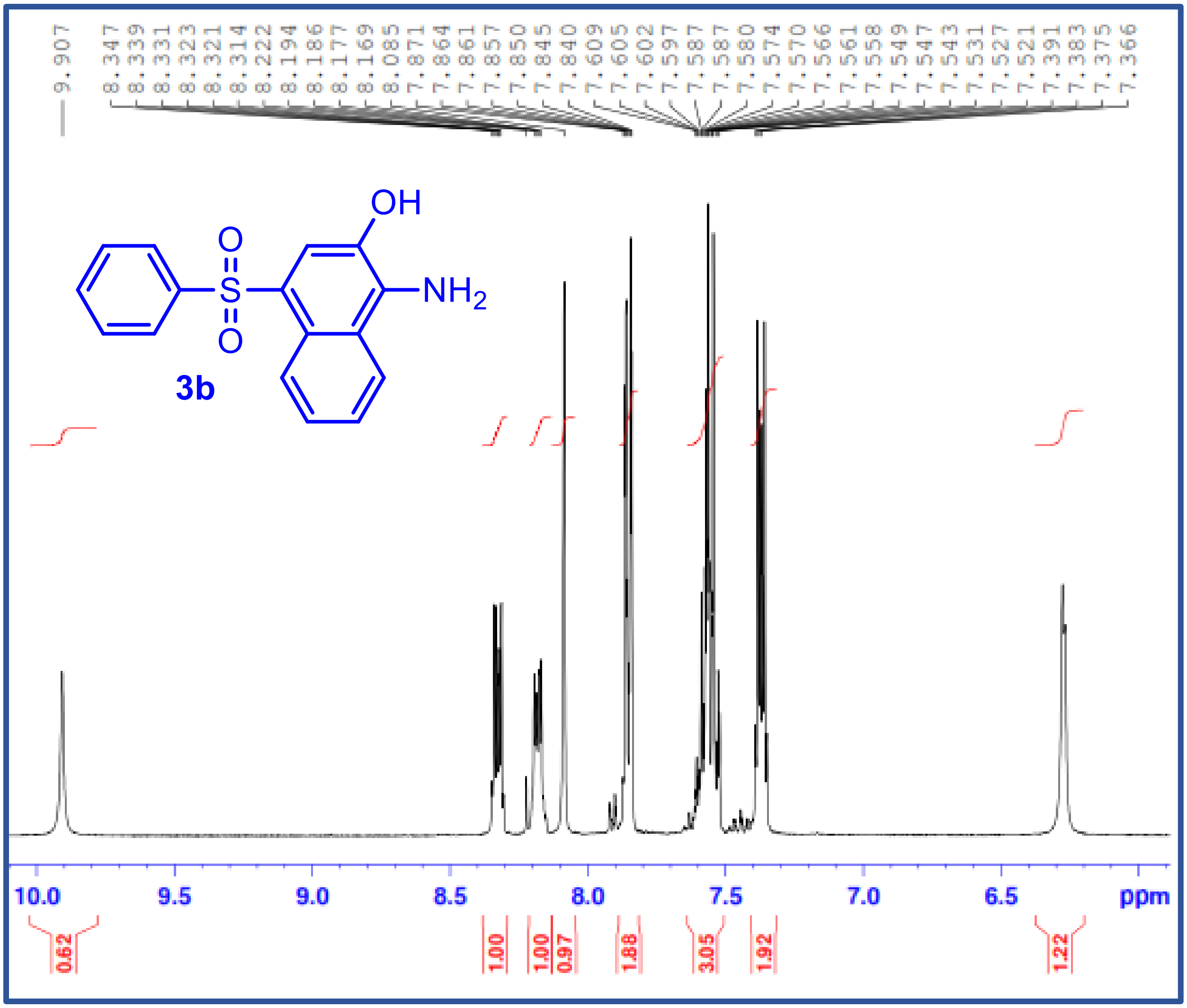

- ^{13}C NMR spectrum of 3b

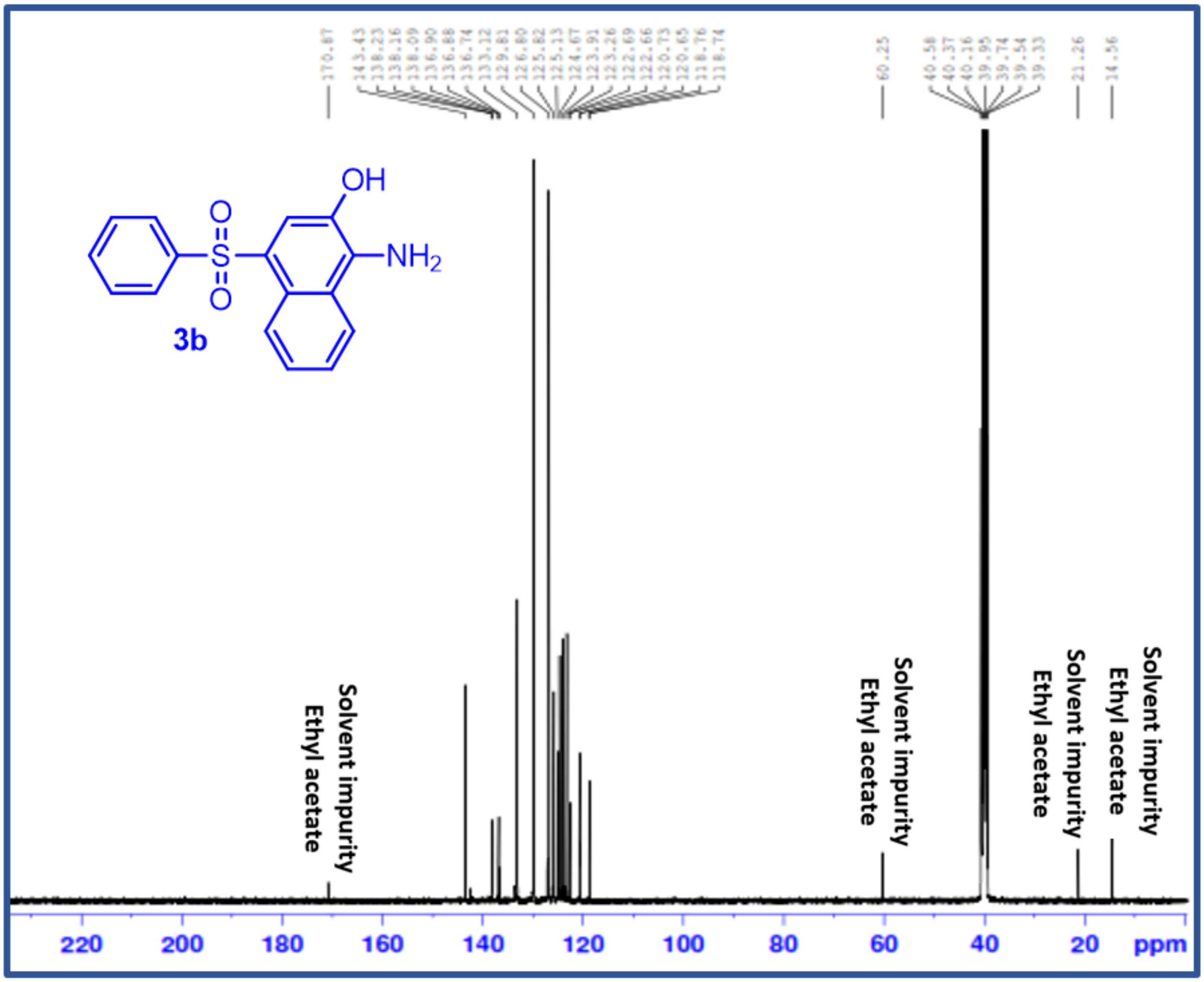

- **MS spectrum of 3b**

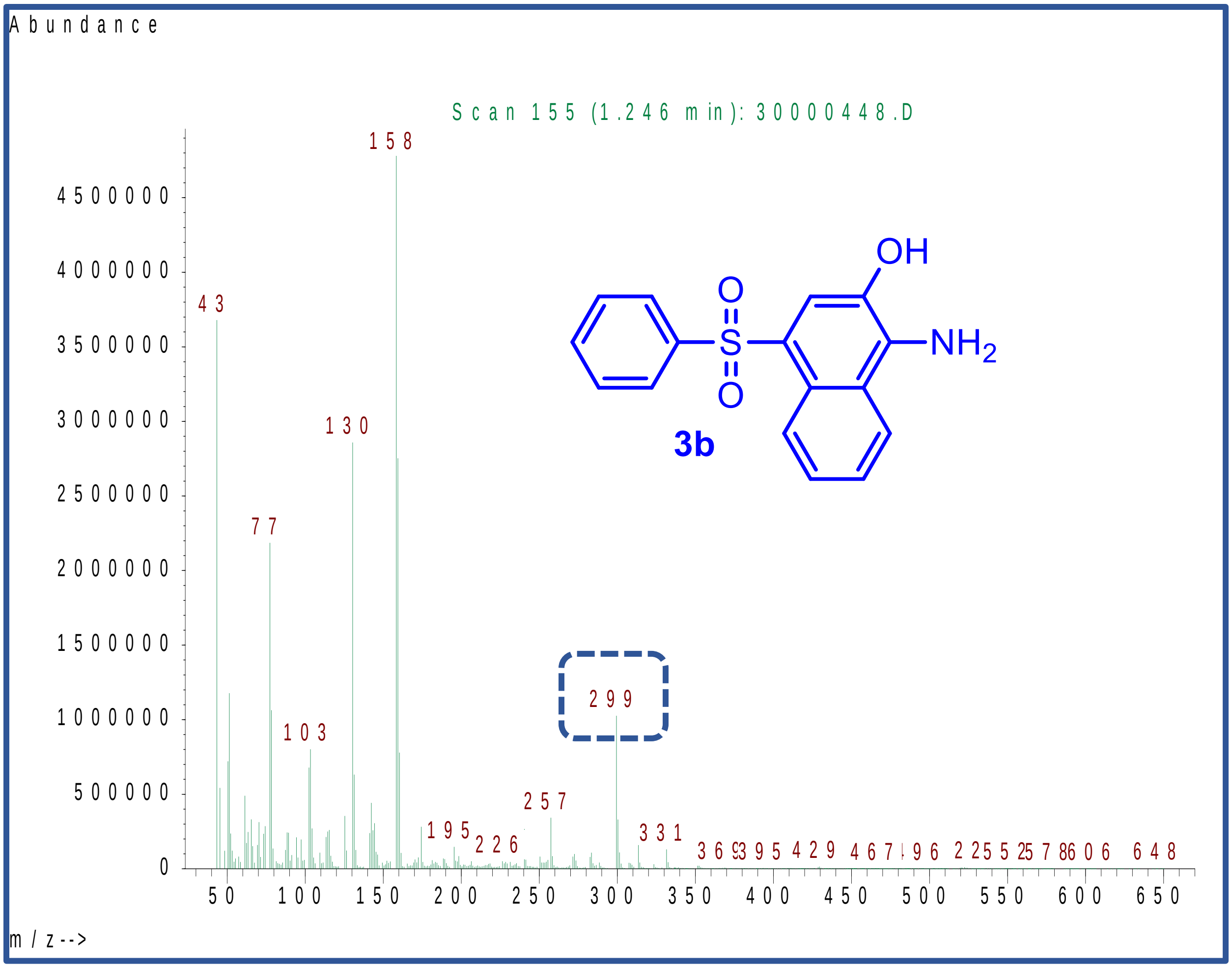

- **IR spectrum of 3c**

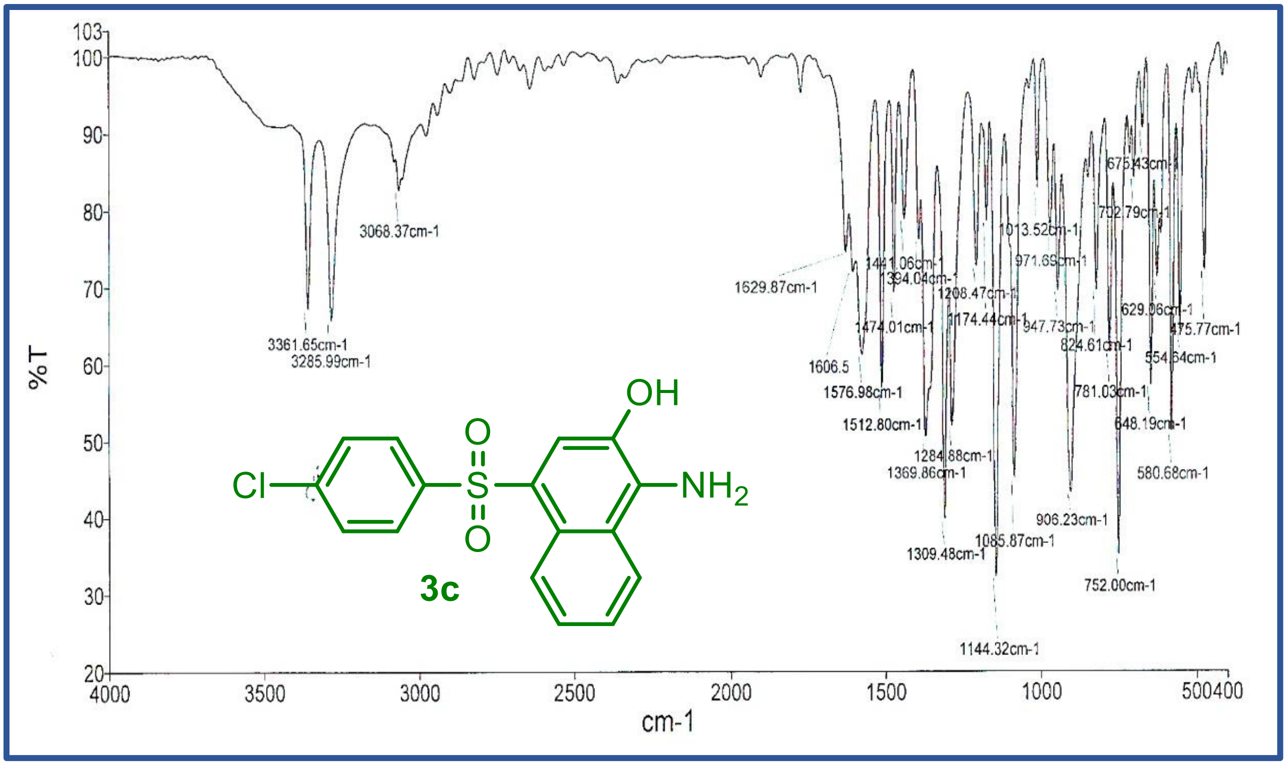

- ^{1}H NMR spectrum of 3c

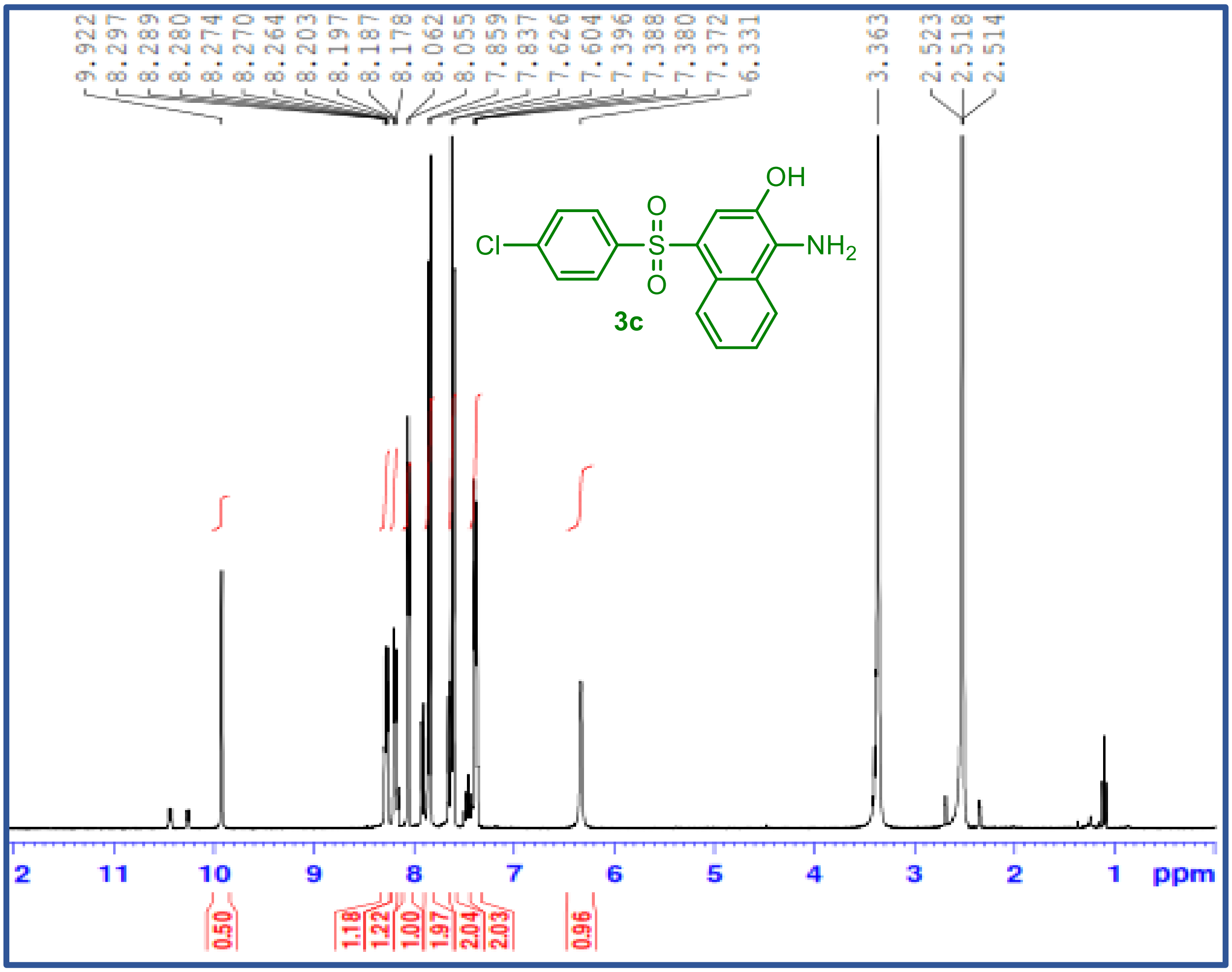

- ^{1}H NMR spectrum of 3c in the presence of D_2O

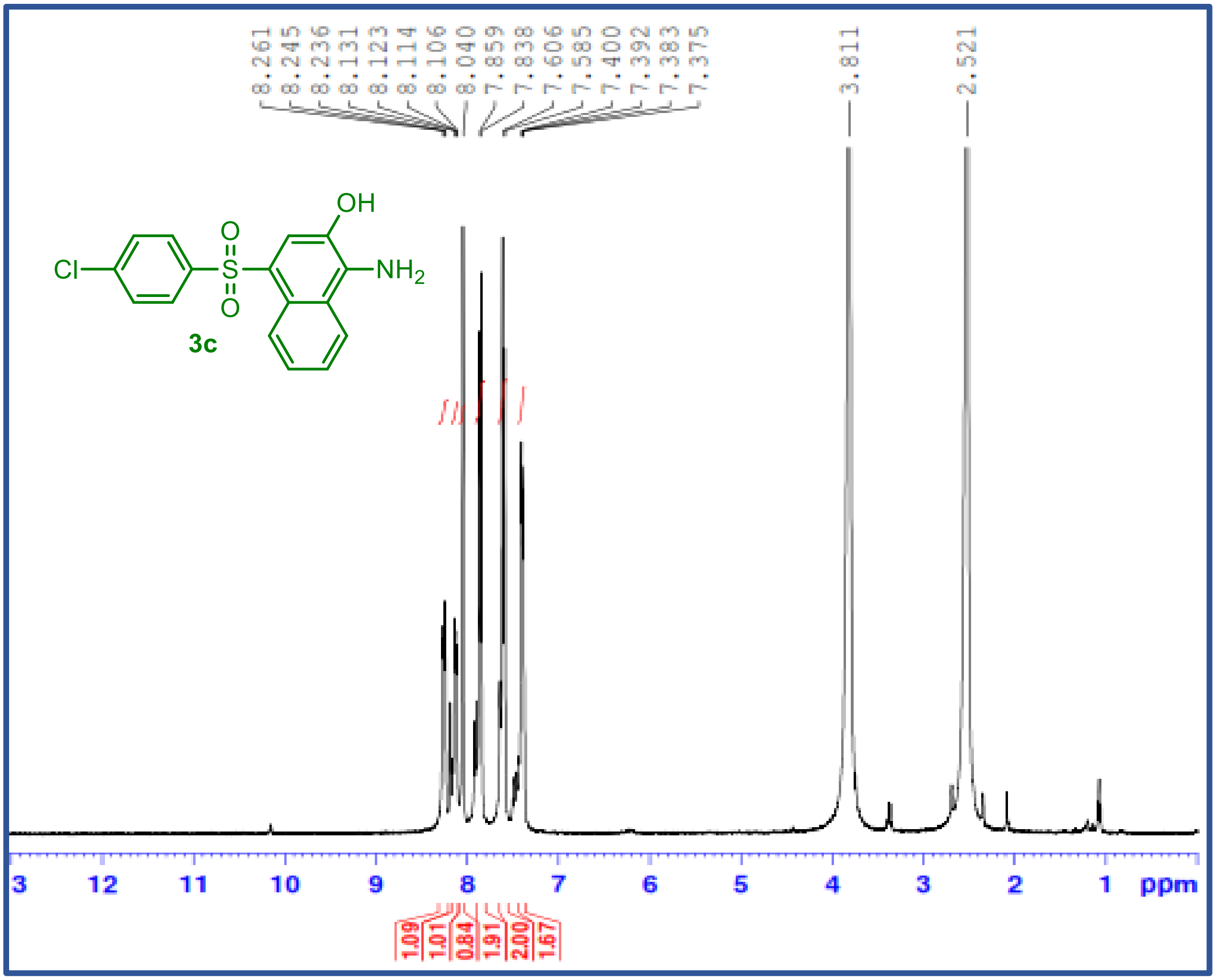

- **Expanded ^{1}H NMR spectrum of 3c**

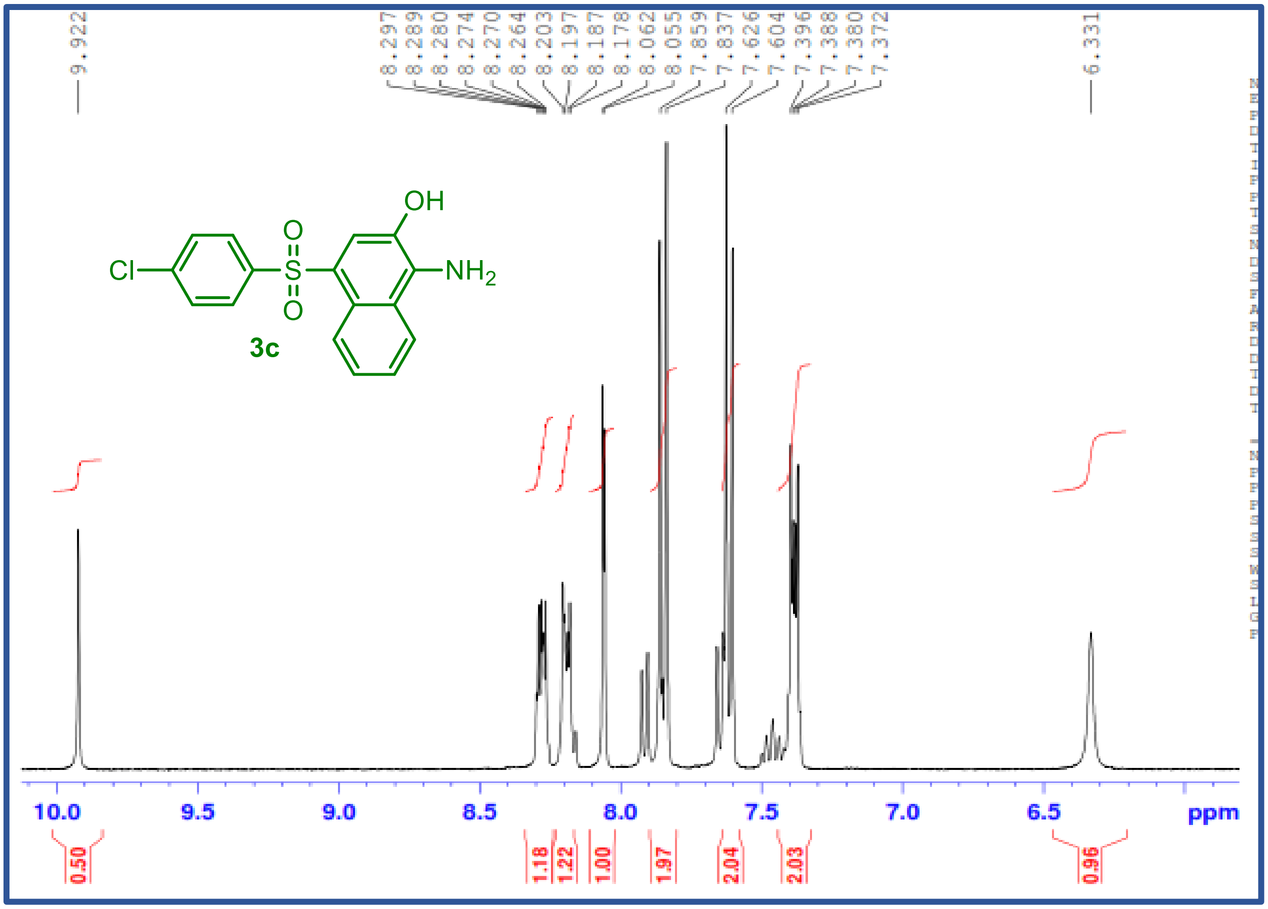

- ^{13}C NMR spectrum of 3c

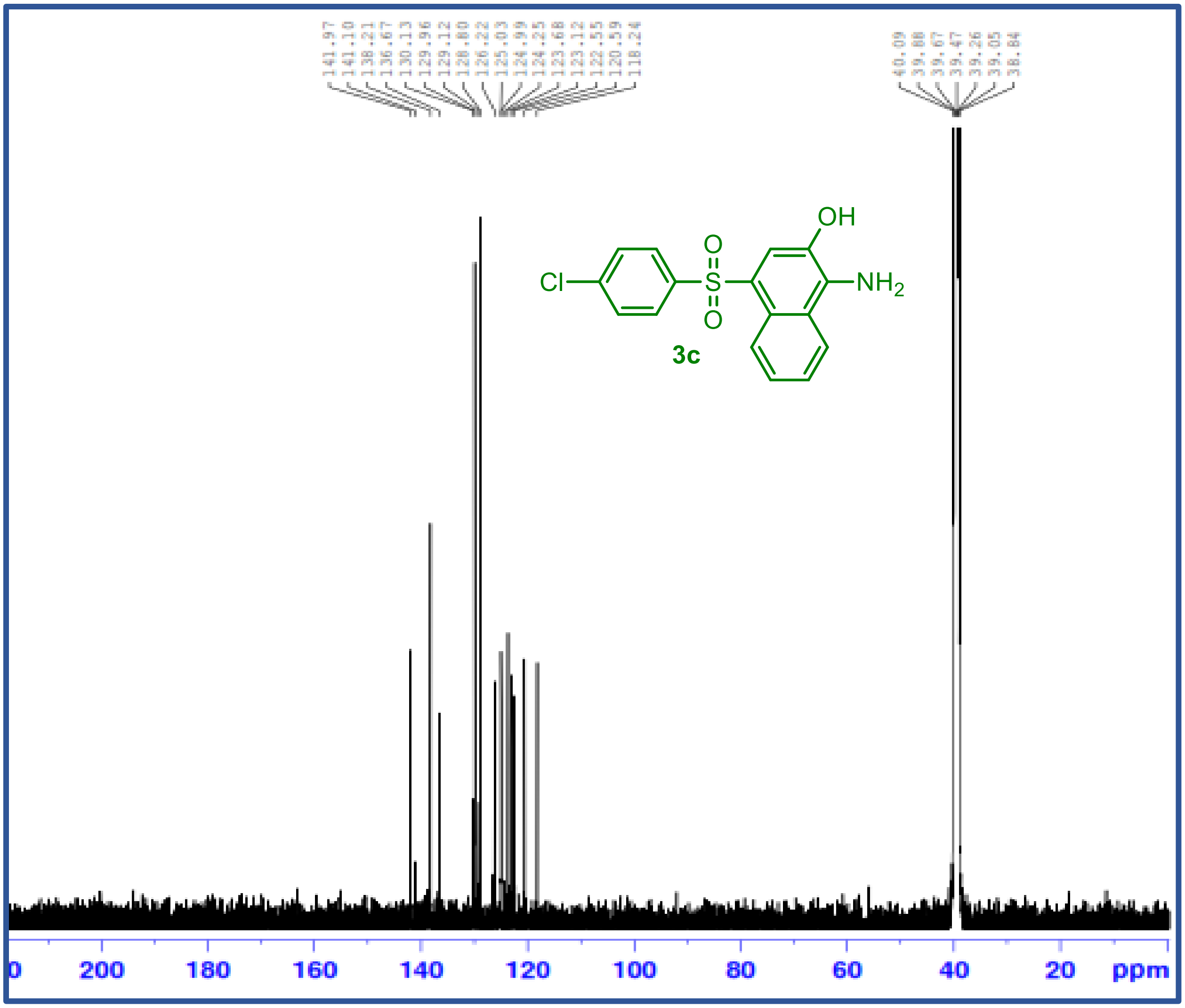

- **MS spectrum of 3c**

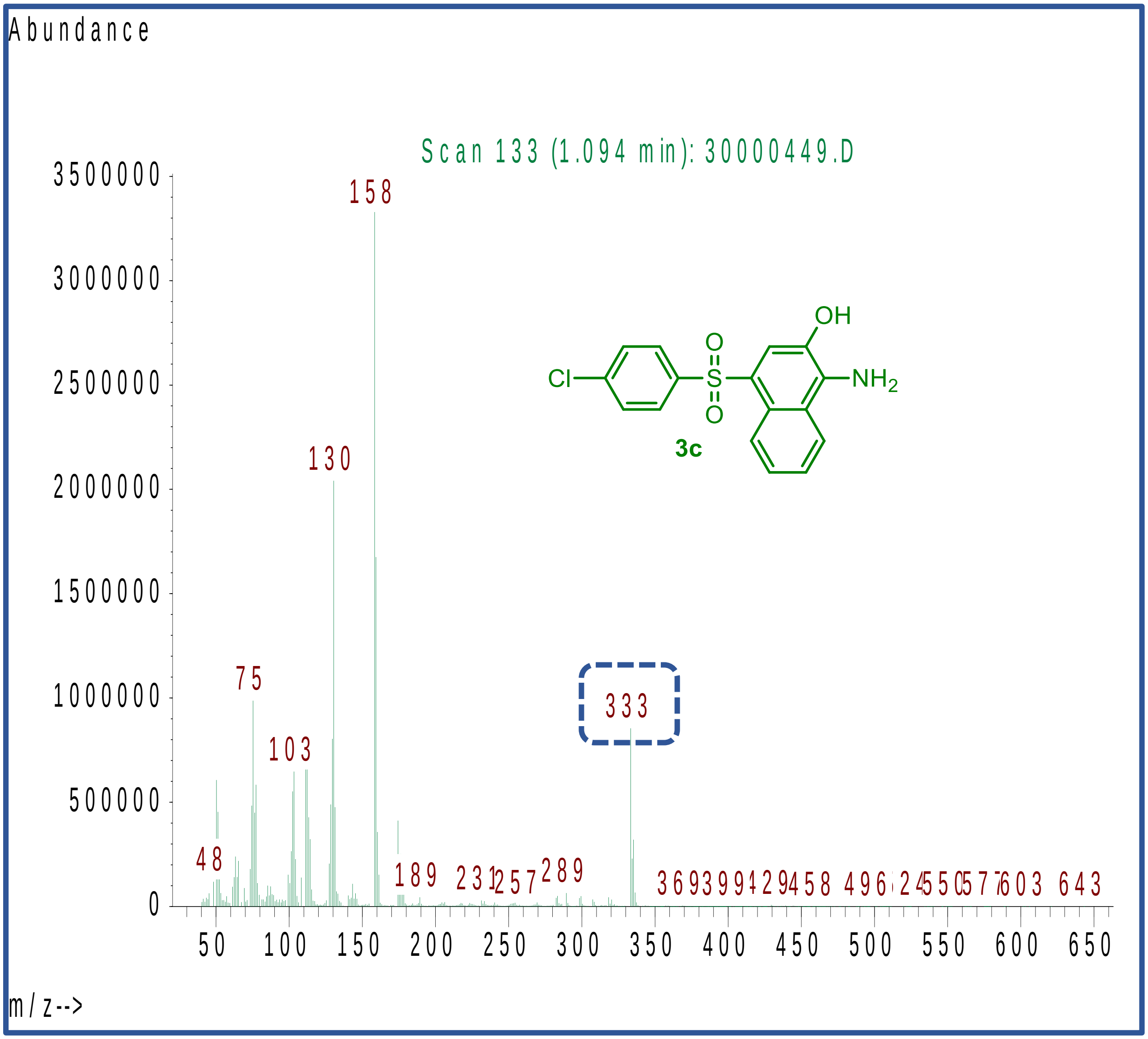

Supplement: Supporting Information [file srep41963-s1.pdf]
